# A new method for fault identification of T-connection transmission line based on multi-scale traveling wave reactive power and random forest

**DOI:** 10.1371/journal.pone.0284937

**Published:** 2023-08-18

**Authors:** Yawen Zhong, Jie Yang, Sheng Wang, Sijing Deng, Liang Hu

**Affiliations:** 1 School of Engineering, Southwest Petroleum University, Nanchong, Sichuan, China; 2 School of Information Engineering, Southwest University of Science and Technology, Mianyang, Sichuan, China; 3 Artificial Intelligence Key Laboratory of Sichuan Province, Sichuan University of Science and Engineering, Zigong, Sichuan, China; 4 State Grid YiliYihe Electric Power Supply Company, Yining, Xinjiang, China; UCSI University Kuala Lumpur Campus: UCSI University, MALAYSIA

## Abstract

Though the traditional fault diagnosis method of T-connected transmission lines can identify the faults inside and outside the area, it can not identify the specific branches. To improve the accuracy and reliability of fault diagnosis of T-connection transmission lines, a new method is proposed to identify specific faulty branches of T-connection transmission lines based on multi-scale traveling wave reactive power and random forest. Based on the S-transform, the mean and sum ratios of the corresponding short-time series traveling wave reactive powers of each two traveling wave protection units at multiple frequencies are calculated respectively to form the fault feature vector sample set of the T-connection transmission line. A random forest fault branch identification model is established, and it is trained and tested by the fault feature sample set of T-connection transmission line to identify the fault branch. The simulation results show that the proposed algorithm can identify the branch where the fault is located inside and outside the protection zone of T-connection transmission line quickly and accurately under various working conditions. This method also shows good performance to identify faults even under the situation of CT saturation, noise influence and data loss.

## 1 Introduction

The safety and stability of the power system operation is the premise to ensure the development of related industries, showing a profound impact on the development of the national economy [1, 2]. For power system, transmission lines, which transmit the electric energy, is one of the most important components. Its fault can influence the power system operation significantly. Power system collapse accidents in domestic and foreign regions are often caused by failure of transmission lines [3–5]. Therefore, it is essential to diagnose faults of transmission lines [6, 7].

T-connection transmission line with high power and heavy load is widely used in high-voltage or ultra-high-voltage power transmission networks to connect the large systems and power plants because of unique wiring characteristics. Large-scale blackouts are often caused by the fault on these lines, causing economic loss. It is necessary to diagnose the locations of faults timely and accurately, and then eliminate the faults [8].

At present, there are two main kinds of methods based on power frequency and transient frequency to diagnose the faults of T-connection transmission line. For power frequency diagnostic method, it relies on the measured information including voltage power frequency, current power frequency and distribution parament of the protection units near the busbar internal and external of T-connection transmission. Sonan et al. [9] analyses the ratio of respective fault component and vector sums of the three-terminal voltage and current to identify the faults. Gao et al. [10] identify the faults based on the sum of the three-terminal current fault components of the vector difference between the maximum current in the three-terminal current fault component and the sum of the currents at the other two. However, the sensitivity and reliability of this method could be influenced by the choice of braking coefficient. To solve this problem, Li et al. [11] uses the maximum current in the three-terminal fault current component of the T-connection transmission line combined with the sum of the currents at the other two and the cosine angle to identify the faults. According to Reference [10, 11], although Wang et al. [12] established a comprehensive criterion to identify of faults inside and outside the PV T-connection high-voltage distribution network area, this paper still not analyses the performance of the algorithm. Nayak et al. [13] calculates the positive sequence voltage at the T node at the three terminals, and compare the maximum amplitude *g*_1_ of the positive sequence voltage superimposed component of the T node and the maximum amplitude *g*_2_ of the positive sequence voltage superimposed component of the three terminals to identifies faults. It is diagnosed as an in-zone failure occurred when *g*_1_ > *g*_2_; or as an out-of-zone failure occurred when *g*_1_ < *g*_2_. Gaur et al. [14] relies on the maximum value of the positive sequence superimposed voltage of the T node calculated by the three terminals of the T-connection transmission line to identify whether a fault occurs. Then proposes the phase *θ* between the positive sequence superimposed voltage and current at a specific terminal to identify the fault inside and outside the protection zone. When *θ* ∈ (0°, 180°), it is diagnosed as a failure occurred in external zone; when *θ* ∈ (180°, 360°), it is diagnosed as a failure occurred in the internal zone. Zheng et al. [15] uses information such as voltage amplitude difference of three sides of T-connection transmission line, measured impedance characteristics, combined voltage amplitude difference and the auxiliary criterion of adaptive distance to identify faults. On the basis of the original current longitudinal differences, Liu et al. [16] establishes auxiliary criterion to identify the faults in the zone of T-type distributed power supply applied in the power distribution network at both ends. But this only relies on the these information including original voltage and current transformers at both ends of the high-voltage transmission, the positive-sequence compensation voltage difference at both ends of the line, and the phase relationship between the positive-sequence compensation voltage and the positive-sequence differential current. And this algorithm ignores the influence of transient control of distributed power supply. Wang et al. [17] adopts a new current differential protection method based on current research results of current differential protection of double-ended lines. This paper proposed an identification method for T-connection transmission line based on distributed parameter model. To conclude, the fault diagnosis method based on power frequency cannot realize the rapid identification due to the long calculation data window. And its sensitivity of fault diagnosis is also easily affected by other variables.

As for methods based on transient quantity, the voltage (current) traveling wave information were processed to identify faults. Kumar et al. [18] estimates the instantaneous value of voltage and current signal phasor by providing the voltage and current signals measured at the relay end of T-connection transmission line to the second-order Taylor Kalman Fourier filter, and then identify faults inside and outside the protection zone by calculating the positive sequence impedance obtained through the instantaneous voltage and current phasor information. Zheng et al. [19] used the cosine similarity of the three-terminal transient currents of T-connected transmission line to establish the criterion to identify faults inside and outside the zone. Although wavelet transform is applied to fault identification of T-shaped transmission line in the literature [20, 21], high-frequency noise signals will affect the effect of fault identification. Bhalja et al. [20] uses bior3.1 wavelet to process the original current signal of the three terminals of the T-connection transmission line, and compares the relationship between the three-terminal operating current and suppression current then to identify. Eissa et al. [21] compares the fault current polarity detected at each end of the T-connection transmission line by applying Haar wavelet function to identify the faults inside and outside the zone. In conclusion, the fault diagnosis method based on transient quantity has attracted extensive attention because it can realize fast protection action. But most existing methods do not further analyze the performance of algorithm, and the accuracy of fault diagnosis needs to be improved.

In recent years, the intelligent fault diagnosis method of power system has been extensively studied, but fewer involved the research of intelligent fault diagnosis method applied to T-connection transmission line. Random forest is a self-supervised ensemble learning algorithm, which can accurately predict a single problem through the integration of multiple trees. It has excellent performance in training speed, generalization ability and dealing with unbalanced data sets. In order to overcome the shortcomings of the traditional T-connection transmission line fault identification algorithm, our paper draws on the research ideas of reference [22–24], analyzes the electrical characteristics of the T-connection transmission line fault, and conducts relevant research on its fault diagnosis method. A new fault identification method for T-connection transmission line based on multi-scale traveling wave reactive power and random forest is proposed. The algorithm calculates the mean sum of the initial traveling wave reactive power by collecting the initial voltage and current traveling waves of multiple short-time sequences at three end measuring points in the T-connection transmission line area after S-transform, compares the mean sum of the reactive power obtained by each two traveling wave protection units corresponding to the short-time sequence to form the T-connection transmission line fault feature vector sample set, which is trained and tested in combination with the random forest fault branch identification model, so as to realize the identification of the T-connection transmission line fault branch. The simulation results show that the algorithm can accurately and sensitively identify the fault branch under various working conditions. The contributions of this article are as followings:

1. This algorithm combines the analysis of fault power distribution characteristics inside and outside T-connected transmission lines and S-transform of a multi-scale reactive power fault characteristic sample set, enriching the fault representation forms of T-connected transmission lines.
2. The neural network is added into the fault diagnosis of T-connected transmission line to improve the stability of fault diagnosis by using the powerful pattern recognition ability of neural network.
3. Compared with traditional fault diagnosis method of T-connected transmission line [9–21], the proposed algorithm can identify the specific branches of the faults inside and outside the area accurately even with the influence of CT saturation, data loss, noise and other factors.

This paper is structured as followings: Section 2 analyses the initial traveling wave power distribution when the T-connected transmission line is located inside or outside of the fault area; Section 3 introduces the related theory and the algorithm flows; Section 4 shows the test and analysis process of the simulation experiment; Section 5 analyzes the superiority and robustness of the algorithm; Section 6 summarizes the full text.

## 2. Analysis of the initial traveling wave power of the faulty

**Fig 1:**
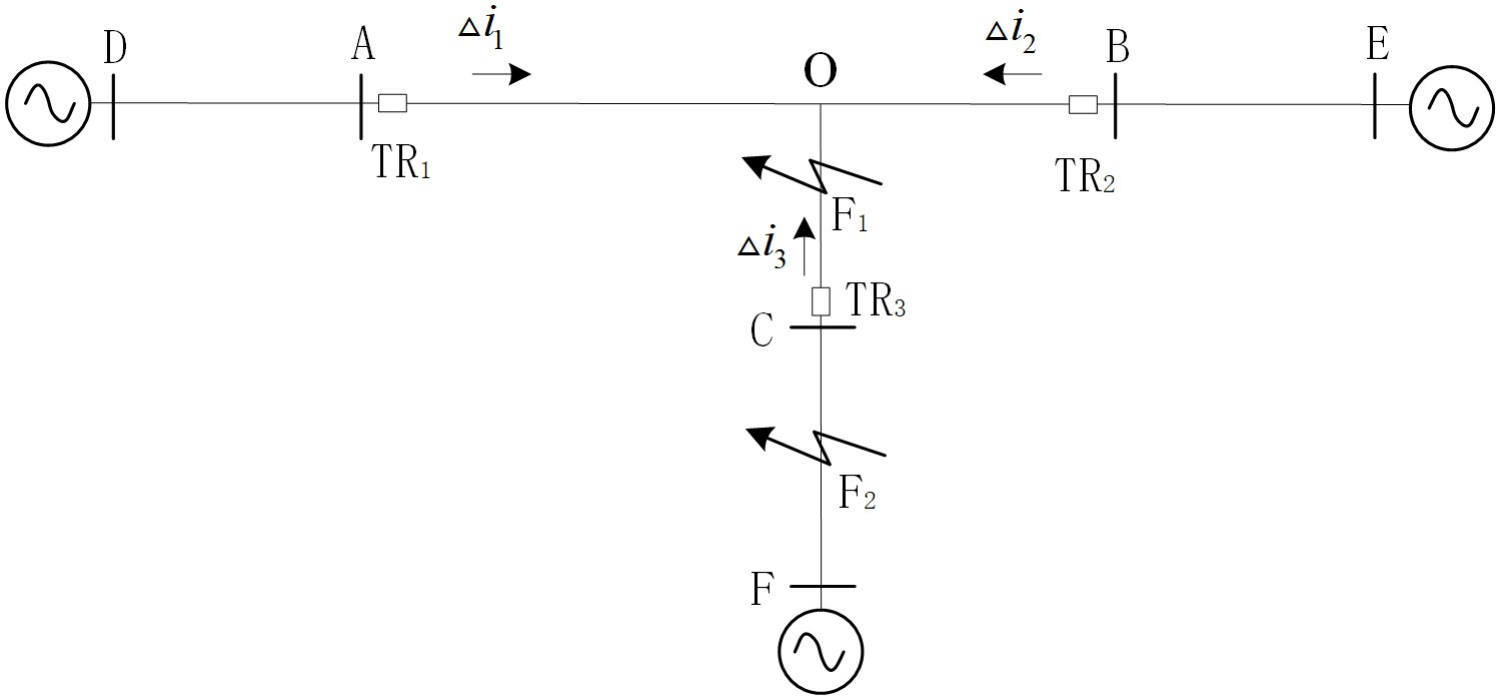
500kV T-Connection transmission line.

As shown in Fig 1, the T-connection transmission line (voltage level is 500 kV) is composed of branches named AO, BO, CO in the internal zone and branches named AD, BE, CF in the external zone. The traveling wave protection unit TR_1_~ TR_3_ is installed in the branches which are near the terminal busbars named A, B, C respectively. When the fault occurs at the AO branch in the protection zone, the traveling wave propagates from the fault point to both sides along the line, and refraction occurs at the discontinuity of the wave impedance of the line [25, 26].

According to the traveling wave propagation theory, the assumptions are as following: *t*_0*m*_ (*m* = 1, 2, 3) is the initial traveling wave reaches terminals A, B, and C for the first time, respectively. *t*_1*m*_ (*m* = 1, 2, 3) is the second time that the traveling wave reaches the three terminals A, B, and C after the refraction occurs at the discontinuous impedance of the traveling wave. During the time period *t*_0*m*_ ~ *t*_1*m*_, the fault traveling waves obtained by the traveling wave protection units *TR*_*m*_ (*m* = 1, 2, 3) near the A, B, and C ends of the branch circuits in the protection zone are called initial voltage traveling waves Δ*u*_*m*_ (*m* = 1, 2, 3) and initial current traveling waves Δ*i*_*m*_ (*m* = 1, 2, 3).

### 2.1 Initial traveling wave power distribution of fault in the protection zone

Assumed that the current polarity of outgoing bus is positive and of incoming bus is negative. The positive and negative power can be judged according to the current polarity of associated lines of each busbar.

**Fig 2:**
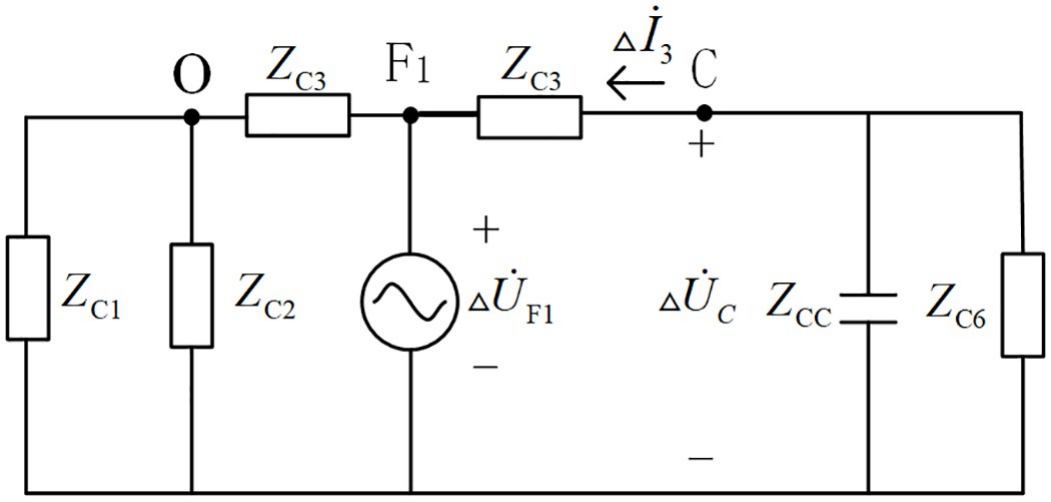
Peterson equivalent circuit when internal branch OC occur fault in T-connection transmission line.

When the fault occurs at *F*_1_ of the branch named CO in the T-connection transmission line protection zone, the Peterson equivalent circuit of the T-connection transmission line is shown in Fig 2. $△{\dot{U}}_{F1}$ is the additional network voltage at the fault point, and $△{\dot{U}}_{C}$ and $△{\dot{I}}_{3}$ are the initial voltage and current traveling waves of the measured bus A, respectively. The wave impedances of lines named OA, OB, OC, AD, BE, CF are *Z*_*C*1_~*Z*_*C*6_ respectively. According to the fact that the line wave impedance is approximately a real constant [27], and the equivalent capacitance impedance of bus C to ground is *Z*_*CC*_.

According to the definition of the initial traveling wave complex power [24], formula of the initial traveling wave complex power at the C terminal of the line bus is:

$$△{\tilde{S}}_{C}=△{\dot{U}}_{C}△{\dot{I}}_{3}^{*}$$ (1)

When a fault occurs on OC in the protection zone, it can be known from the Peterson equivalent circuit in Fig 2 that:

$$△{\dot{I}}_{3}=-\frac{△{\dot{U}}_{C}}{Z_{C6}//Z_{CC}}$$ (2)

The complex power measured by the *TR*_3_ traveling wave protection unit at terminal C:

$$△{\tilde{S}}_{C}=△{\dot{U}}_{C}\times △{\dot{I}}_{3}^{*}=△{\dot{U}}_{C}\times {\left(-\frac{△{\dot{U}}_{C}}{Z_{C6}//Z_{CC}}\right)}^{*}=-△U_{C}^{2}\times \frac{1}{Z_{C6}//Z_{CC}}=P_{C}+jQ_{C}$$ (3)

In the formula, *P*_*C*_ is the active power of the initial traveling wave of the line, and *Q*_*C*_ is the reactive power of the initial traveling wave of the line. When an internal fault occurs on T-connection transmission line:

$$Q_{C}=-△U_{C}^{2}\times \frac{1}{Z_{C6}//Z_{CC}}<0$$ (4)

### 2.2 Initial traveling wave power distribution of out-of-zone fault

**Fig 3:**
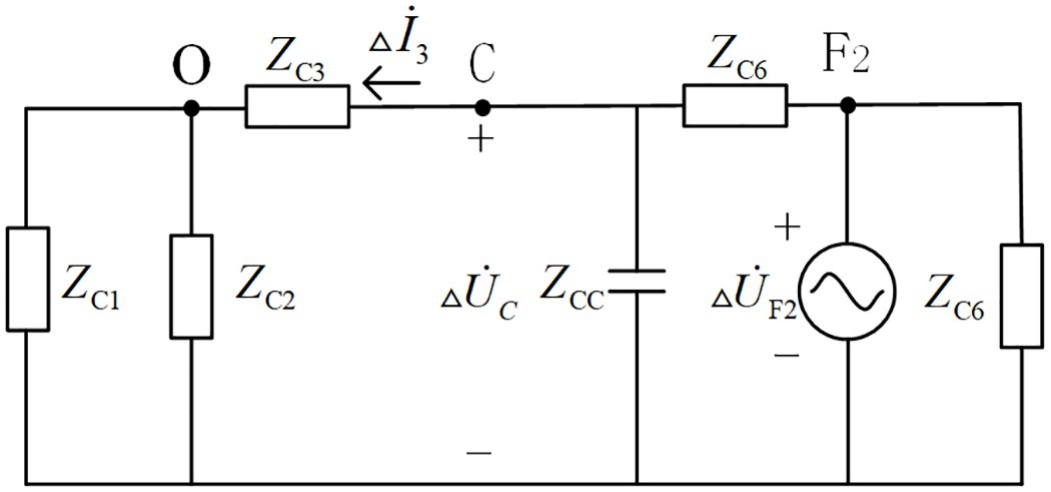
Peterson equivalent circuit when external branch CF occur fault in T-connection transmission line.

When a fault occurs at location named *F*_2_ of CF branch outside the protection zone of the T-connection transmission line, the Petersen equivalent circuit of the T-connection transmission line is shown in Fig 3.

Fig 3 shows that:

$$\Delta {\dot{I}}_{3}=\frac{\Delta {\dot{U}}_{C}}{Z_{CC}+Z_{C1}//Z_{C2}}$$ (5)

Thus, the complex power measured by the traveling wave protection unit *TR*_3_ is:

$$△{\tilde{S}}_{C}=△{\dot{U}}_{C}△{\dot{I}}_{3}^{*}=△{\dot{U}}_{C}\times \frac{△{\dot{U}}_{C}^{*}}{Z_{C3}+Z_{C1}//Z_{C2}}=△U_{C}^{2}\times \frac{1}{Z_{C3}+Z_{C1}//Z_{C2}}=P_{C}+jQ_{C}$$ (6)

In the formula, *P*_*C*_ is the active power of the initial traveling wave of the line, *Q*_*C*_ is the reactive power of the initial traveling wave of the transmission line, when a fault occurs outside the protection zone of the T-connection transmission line:

$$Q_{C}=\frac{△U_{C}^{2}}{Z_{C3}+Z_{C1}//Z_{C2}}>0$$ (7)

## 3 Related theory and process of algorithm

In the three-phase transmission system, the coupling between voltage and current of each phase will affect the voltage and current. It is necessary to decouple the voltage and current of each phase. In this paper, Clark phase mode transformation is used to decouple the voltage and current of each phase. And then the combined modulus method is used to reflect various fault types of T-connection transmission line [24, 27],

$$\left\{\begin{array}{l} \Delta u_{z}=4\Delta u_{\alpha }+\Delta u_{\beta }\\ \Delta i_{z}=4\Delta i_{\alpha }+\Delta i_{\beta } \end{array}\right.$$ (8)

Where Δ*u*_*α*_ and Δ*u*_*β*_ represent the Clark *α* and *β* mode voltages, respectively; Δ*i*_*α*_ and Δ*i*_*β*_ represent the Clark *α* and *β* mode currents, respectively.

Because S transform method has good ability to extract signal feature in time-frequency analysis. Referring to reference [28], discrete S-transform method is used to convert current and voltage traveling wave moduli of decoupling fault. Then complex matrix reflecting the time-frequency characteristics of current and voltage signals were obtained after S transform. It is noted that the rows of the matrix are from the frequency information of the traveling wave after discrete S-transform, and the columns of the matrix are from the amplitude information and phase information of each sampling time point in the traveling wave time. Select the information of sampling points near the initial traveling wave head of current and voltage at multiple frequencies after S transform, and calculate the initial traveling wave reactive power of each sampling point.

### 3.1 S-transform principle

S-transform is an extension of wavelet transform and short-time Fourier transform. The choice of window function is avoided, and the defect of fixed window width is improved. At the same time, the feature quantity extracted by S-transform are insensitive to noise [29]. The continuous S-transform of a time signal *h*(*t*) is defined as:

$$S(\tau ,f)=\int_{-\infty }^{\infty }h(t)g(\tau -t,f)e^{-i2\pi ft}dt$$ (9)

$$g(\tau -t,f)=\frac{\left|f\right|}{\sqrt{2\pi }}e^{-\frac{{\left(\tau -t\right)}^{2}}{2\sigma^{2}}}$$ (10)

Among them, *g*(*τ*-*t*, *f*) represents the Gaussian window function; *τ* represents the parameter that controls the position of the window function on the time axis; *f* represents the frequency; *σ* = 1/|*f*|.

The discrete time series *h*[*kT*](*k* = 0, 1, 2,⋯, *N* − 1) is obtained by sampling the signal *h*(*t*), where T is the sampling interval and N is the number of sampling points, then the discrete Fourier transform function of *h*[*kT*] is:

$$h[\frac{n}{NT}]=\frac{1}{N}\sum_{k=0}^{N-1}h[kT]e^{-j\frac{2\pi kn}{N}}$$ (11)

In the formula, *n* = 0, 1,⋯, *N* − 1.

Then the discrete S transform of the signal *h*(*t*) is:

$$S[kT,\frac{n}{NT}]=\sum_{r=0}^{N-1}H(\frac{r+n}{NT})e^{-\frac{2\pi^{2}r^{2}}{n^{2}}}e^{{}^{j\frac{2\pi rk}{N}}},n\neq 0$$ (12)

$$S[kT,0]=\frac{1}{N}\sum_{r=0}^{N-1}h(\frac{r}{NT}),n=0$$ (13)

After transforming the signal S, a complex matrix reflecting the time-frequency characteristics of the signal is obtained. After S-transform, a two-dimensional time-frequency matrix is obtained. The rows of the matrix correspond to the frequency information of the traveling wave after discrete S-transform, and the columns of the matrix correspond to the amplitude and phase information of each sampling time point in the traveling wave time domain.

### 3.2 Initial traveling wave reactive power analysis of faults of the T-connection transmission line

#### 3.2.1 Analysis of initial traveling wave reactive power of faults occurred in the internal zone

**Fig 4:**
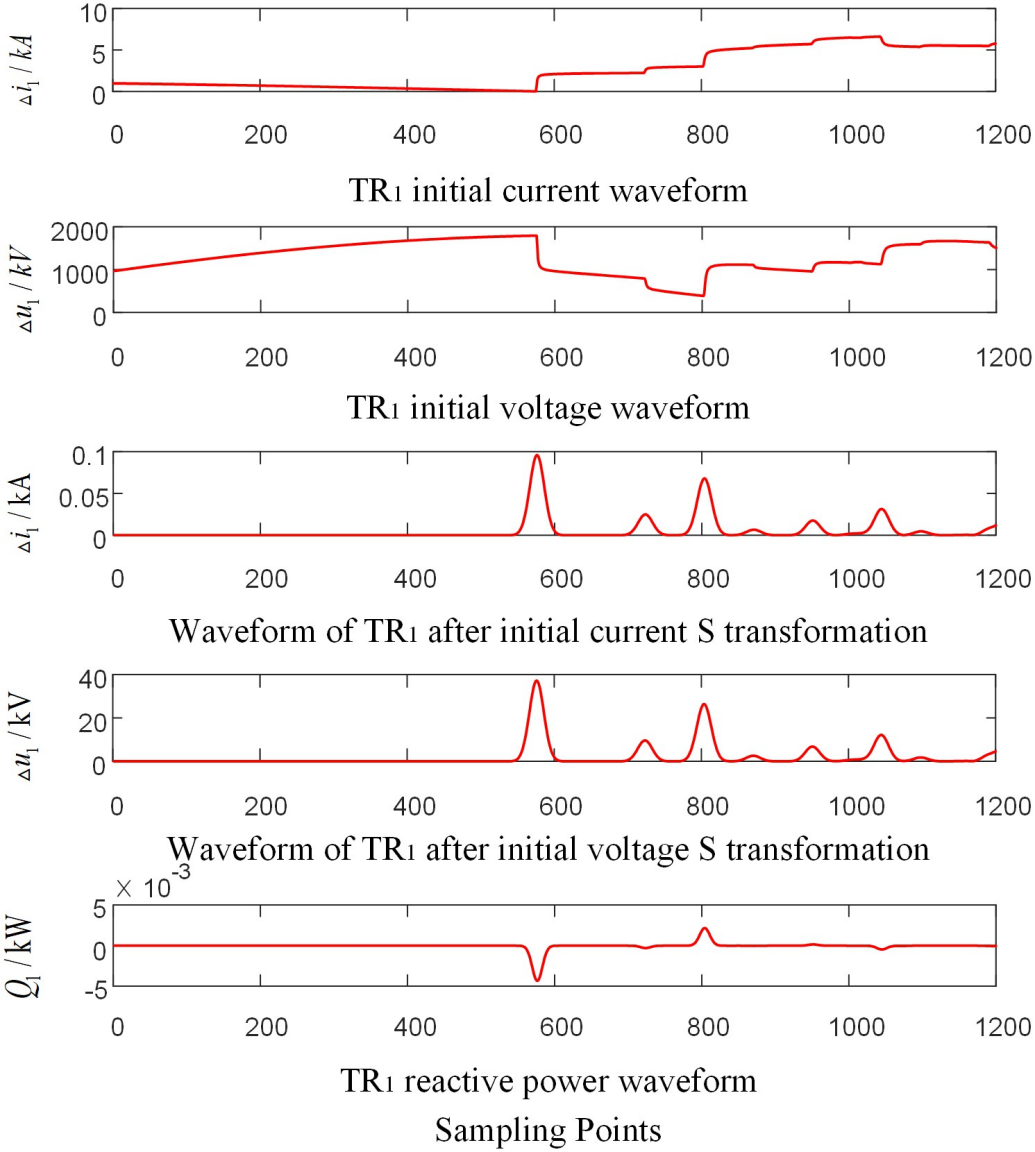
Corresponding waveform diagram of traveling wave protection unit *TR*_1_ when internal branch CO occur fault.

**Fig 5:**
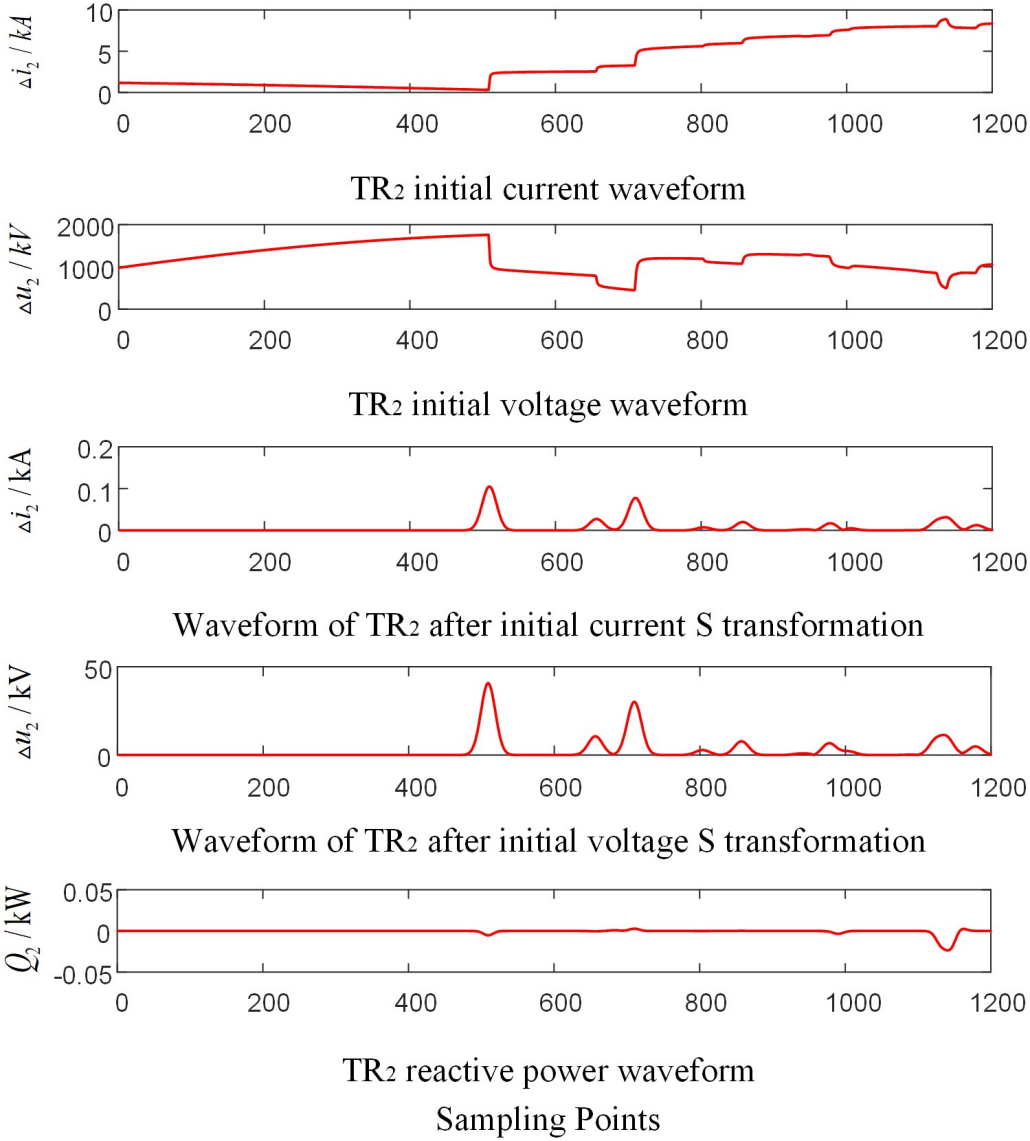
Corresponding waveform diagram of traveling wave protection unit *TR*_2_ when internal branch BO occur fault.

**Fig 6:**
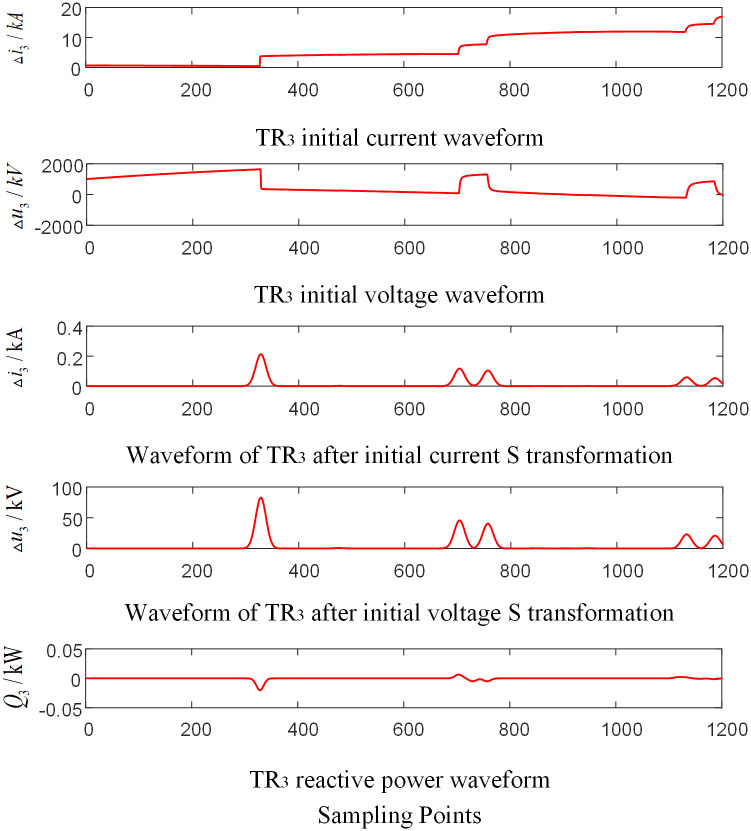
Corresponding waveform diagram of traveling wave protection unit *TR*_3_ when internal branch CO occur fault.

Assumed that the AC phase-to-ground fault occurs at the CO branch 110km away from the O point in the zone of T-connection transmission line. And the initial fault Angle is 60 degrees and the transitional resistance is 100Ω. Taking the signal corresponding to the frequency of 15kHz after S-transformation as an example, the corresponding waveforms of each traveling wave protection unit *TR*_*m*_ are shown in Figs 4–6 respectively, where, △*i*_*m*_ and △*u*_*m*_ are the initial traveling current and voltage traveling wave of *TR*_*m*_ (*m* = 1, 2, 3), respectively. *Q*_*m*_ is the initial traveling wave reactive power distribution waveform of the traveling wave protection unit *TR*_*m*_ (*m* = 1, 2, 3).

As shown in Figs 4–6, when a fault occurs in CO branch of the T-connection line area, the reactive power values were obtained by multiplying the data near head of traveling wave of initial voltage and current from the traveling wave protection unit *TR*_*m*_ (*m* = 1, 2, 3). The results are negative.

#### 3.2.2 Analysis of initial traveling wave reactive power of out of zone fault

**Fig 7:**
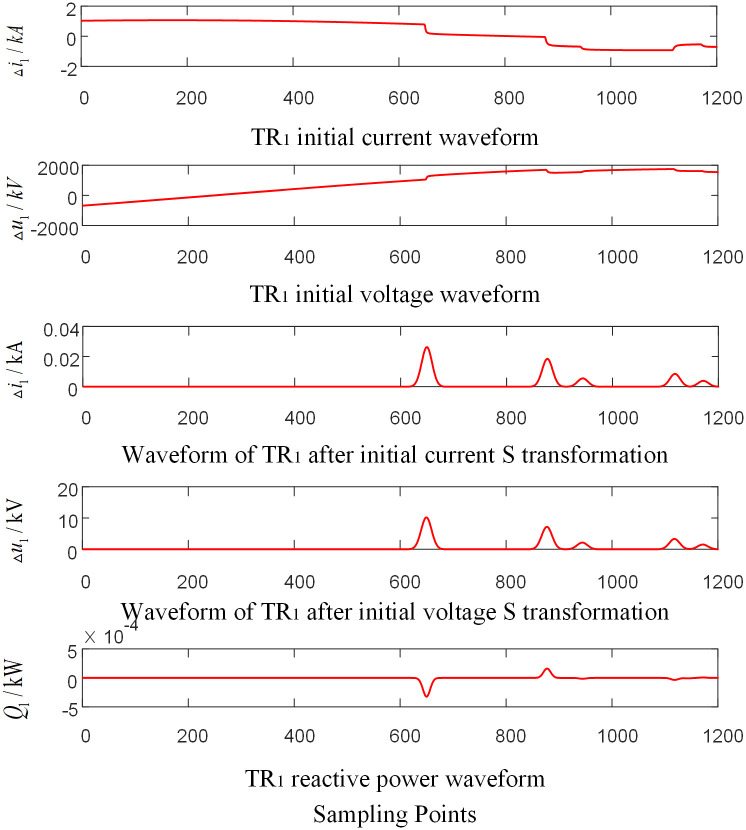
Corresponding waveform diagram of traveling wave protection unit *TR*_1_ when external branch CF occur fault.

**Fig 8:**
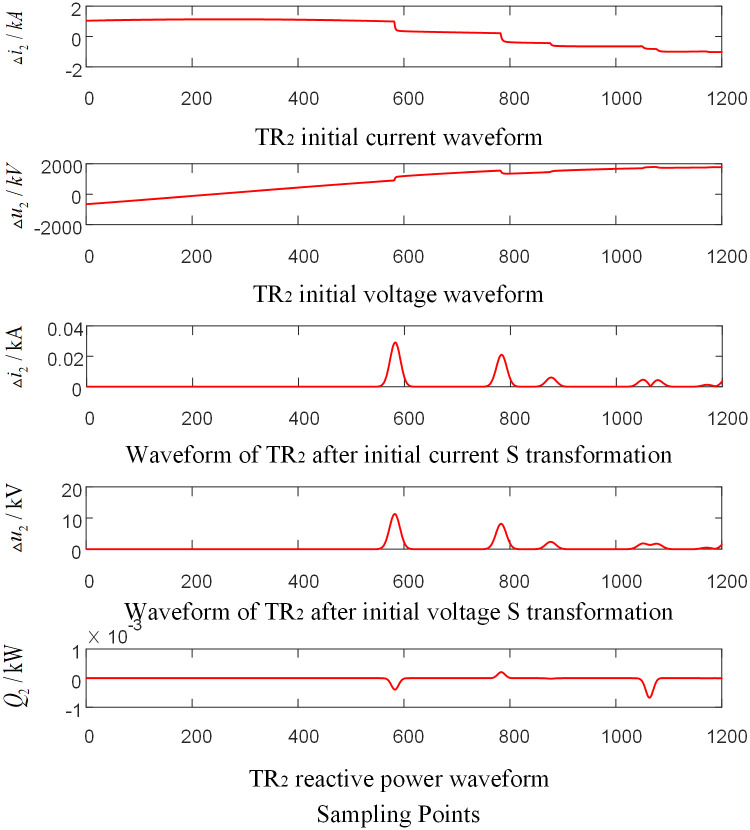
Corresponding waveform diagram of traveling wave protection unit *TR*_2_ when external branch CF occur fault.

**Fig 9:**
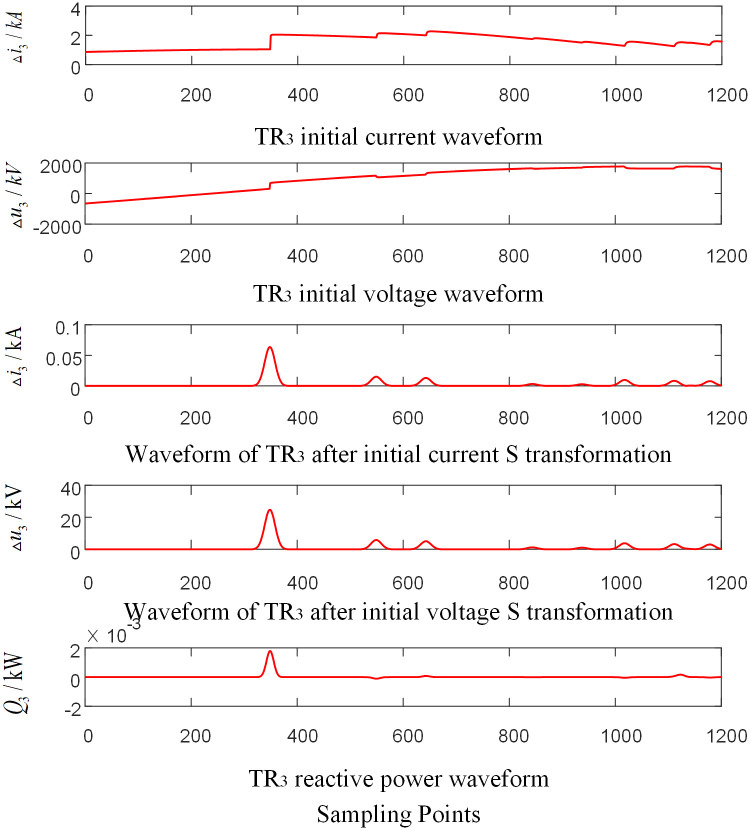
Corresponding waveform diagram of traveling wave protection unit *TR*_3_ when external branch CF occur fault.

Assumed that a BC phase-to-phase failure occurred 220 km away from the O point on the branch CF outside the zone of the T-connection transmission line, the initial fault angle is 5°, and the transitional resistance is 350 Ω. The corresponding waveforms of each traveling wave protection unit are shown in Figs 7–9 respectively (taking the signal corresponding to 15KHz frequency after s transformation as an example).

As shown in Figs 7–9, when a fault occurs on the Branch CF in the protection zone of T-connection transmission line, the reactive power values are obtained from the data near the wave head of the initial voltage and current traveling wave of the traveling wave protection unit *TR*_3_.The results are positive. And the reactive power values obtained from the data near the wave head of the initial voltage and current traveling wave of the traveling wave protection unit *TR*_*m*_ (*m* = 1, 2) are negative.

### 3.3 Calculation of initial traveling wave reactive power after S-transform

Perform S-transformation on the voltage and current traveling waves from each traveling wave protection unit *TR*_*m*_ (*m* = 1, 2, 3) combination of the T-connection transmission line respectively. After S transformation of initial voltage traveling wave and initial current traveling, select the phasors $△{\dot{U}}_{mn}\left(l\right)$, and $△{\dot{I}}_{mn}\left(l\right)$ corresponding to *l* sampling points of wave within 0.1ms after fault occurs at 10 different frequencies *f*_*n*_ (*n* = 1,2,…, 10). And the reactive power *Q*_*mn*_ (*l*) at the corresponding frequency of each sampling point are calculated respectively.

The reactive power *Q*_*mn*_ (*l*) obtained at each frequency is intercepted with 11 sampling points as the fixed data window length and 1 sampling point as the sliding scale factor to obtain ki short-time sequences *Q*_*mnk*_ (*x*)(*k* = 1, 2,⋯, 10; *x* = 1, 2,⋯, 11).The reactive power mean value ${\bar{Q}}_{mn}\left(k\right)$ of *K* short-time sequences intercepted at each frequency under the traveling wave protection unit *TR*_*m*_ is calculated respectively. And then the sum of the average reactive power mean value $Q_{mk}=\sum_{n=1}^{10}Q_{mk}\left(n\right)$ of the k-th short-time sequence corresponding to n frequencies of each traveling wave protection unit is calculated respectively. Finally, the ratio *Q*_12_ = *Q*_1*k*_/*Q*_2*k*_, *Q*_13_ = *Q*_1*k*_/*Q*_3*k*_ and *Q*_23_ = *Q*_2*k*_/*Q*_3*k*_ of the mean sum of reactive power under the corresponding time window k-th sequence of each two traveling wave protection units are calculated. And the ratio of the mean sum of reactive power under the corresponding time window of each two traveling wave protection units is defined as the characteristic vector *Q* of reactive power of T-connection transmission line. Characteristic vector represents the fault characteristics of T-connected transmission lines. Besides, *Q* = [*Q*_12_, *Q*_13_, *Q*_23_]_1×30_.

This section takes the short-term fixed time window sequence reactive power mean value and calculation at a specific frequency *f*_*n*_ of the m-th traveling wave protection unit *TR*_*m*_ as an example. The specific steps are as follows:

1. Perform S-transformation on the initial voltage and current traveling waves measured by the traveling wave protection unit *TR*_*m*_, respectively. This is used to obtain the complex time-frequency matrices of the initial voltage and current traveling waves, which are denoted as matrix *SV*_*m*_ and *S*_Im_ respectively.
2. Select the traveling wave data of 20 sampling points of initial voltage and current corresponding to the frequency *f*_*n*_ after S-transformation, respectively. This is expressed as $△{\dot{U}}_{mn}\left(l\right)$ and $△{\dot{I}}_{mn}\left(l\right)$, where *l* = 1, 2,⋯, 20.
3. Use the following formula (15) to obtain the complex power corresponding to each sampling point at frequency *f*_*n*_:
$$△S_{mn}\left(l\right)=△{\dot{U}}_{mn}\left(l\right)\times △{\dot{I}}^{*}{}_{mn}\left(l\right)=P_{mn}\left(l\right)+jQ_{mn}\left(l\right)$$ (14)
4. Take 11 sampling points as the fixed data window length and 1 sampling point as the slip scale factor for the reactive power *Q*_*mn*_ (*l*) obtained at the frequency *f*_*n*_ to obtain 10 short-time series *Q*_*mnk*_ (*x*) (*k* = 1, 2,⋯, 10; *x* = 1, 2,⋯, 11). And calculate the mean value ${\bar{Q}}_{mn}\left(k\right)$ of reactive power under corresponding short-time sequence, where ${\bar{Q}}_{mn}\left(k\right)=\left(\sum_{x=1}^{11}Q_{mnk}\left(x\right)\right)/11$;
5. Calculate the sum $Q_{m\_k}=\sum_{n=1}^{10}Q_{mk}\left(n\right)$ of mean value of reactive power of the short-time sequence corresponding to the 10 frequencies of each traveling wave protection unit;
6. Finally, calculate the ratio of the mean value sum of reactive power under the corresponding short-time sequence of each pair of traveling wave protection units *Q*_12_ = *Q*_1_*k*_/Q_2_*k*_, *Q*_13_ = *Q*_1_*k*_/Q_3_*k*_, *Q*_23_ = *Q*_2_*k*_/Q_3_*k*_.

### 3.4 Random forest

**Fig 10:**
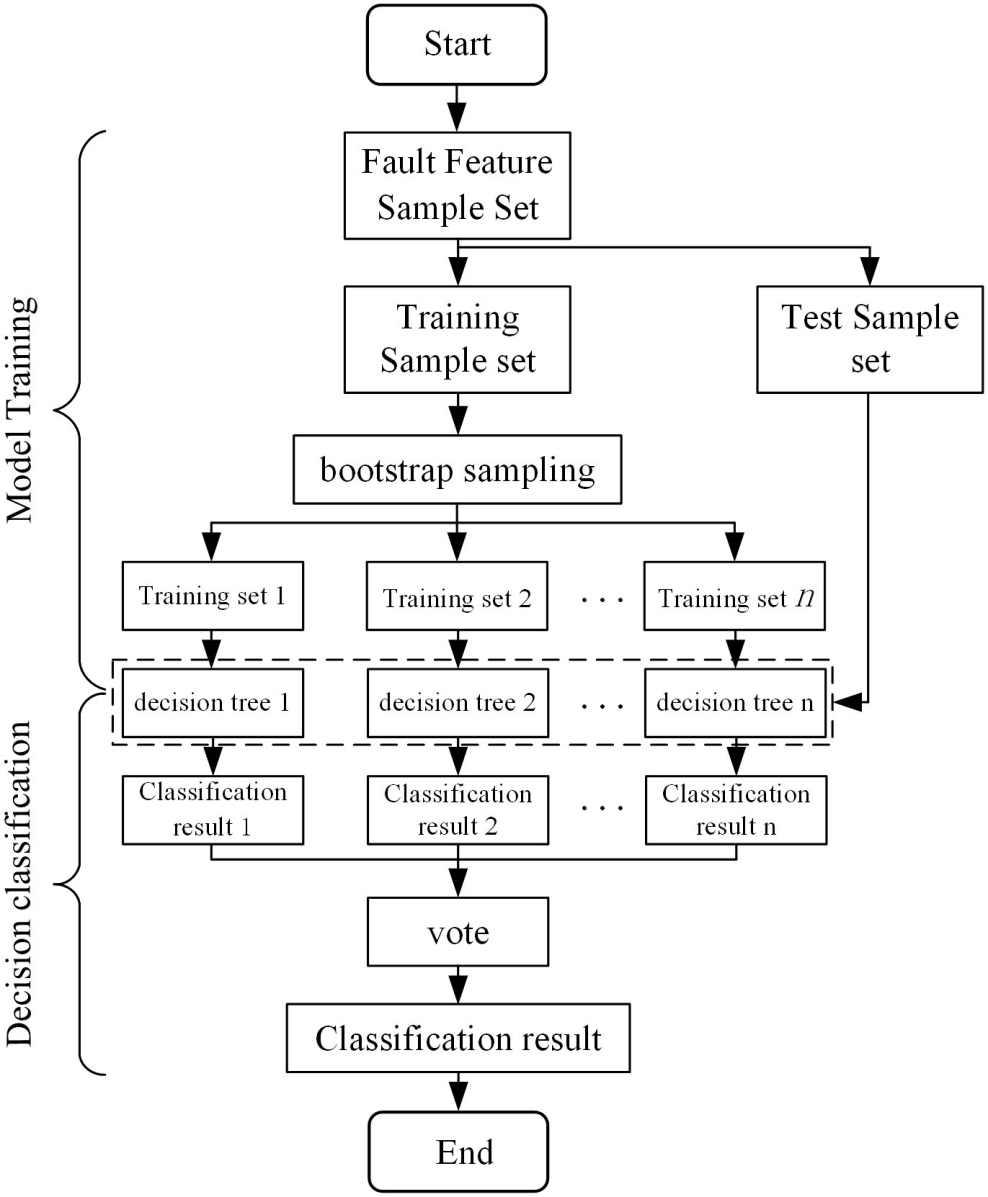
Random forest classification method process.

Random Forest (RF) algorithm is a more efficient and flexible machine learning method based on decision tree [30, 31]. This algorithm integrates multiple trees through the idea of integrated learning [32, 33]. The basic principle relies on Bootstrap resampling technology to extracted N bootstrap data sets from the original data set randomly. Then, N decision trees are trained to form a random forest classifier. Finally, the classification decision is made by voting that the minority is subordinate to the majority. Because the training of decision tree classifier follows the rules of random samples and random features. This method shows good characteristics of strong generalization ability, simple model and good classification effect. Random forest mainly includes two processes: model training and decision classification, and the classification process is shown in Fig 10.

Given a training sample set *X* = {(*x*_1_, *y*_1_), (*x*_2_, *y*_2_),⋯, (*x*_*N*_, *y*_*N*_)}, where x_m_ = [*x*_*m*1_, *x*_*m*2_,⋯, *x*_*mk*_]^*T*^ is the *m*-th training sample, *k* is the number of feature values in *x*_*m*_, and *y*_*m*_ is the class label corresponding to training sample *x*_*m*_.

Model training: bootstrap sampling is performed on the training sample set *X*, and the Bootstrap sub-sample set *X*_*j*_ (*j* = 1, 2,⋯, *n*) is obtained after sampling n times. For each sub-sample set *X*_*j*_, CART is used to build a decision tree model *h*_*j*_(*x*), and finally a classifier {*h*_1_(*x*), *h*_2_(*x*),⋯, *h*_*j*_(*x*)}(*j* = 1, 2,⋯, *n*) composed of a set of decision trees is obtained.

Classification of decision: when the test sample is input into the trained classifier, the category voting is carried out through the established *n* decision trees, and the category with the highest vote is taken as the reddest output category of the test sample. The classification decision is as follows:

$$f\left(x\right)=\text{arg}\;{\text{max}}_{y}\sum_{j=1}^{n}I\left\{\left.h_{j}\left(x\right)=y\right\}\right.$$ (15)

Among them, *h*_*j*_(*x*) is the *j*-th decision tree; *I*{·} is the indicative function, which is 1 when the expression is satisfied, and 0 otherwise; y is the category label.

### 3.5 Fault identification algorithm

**Fig 11:**
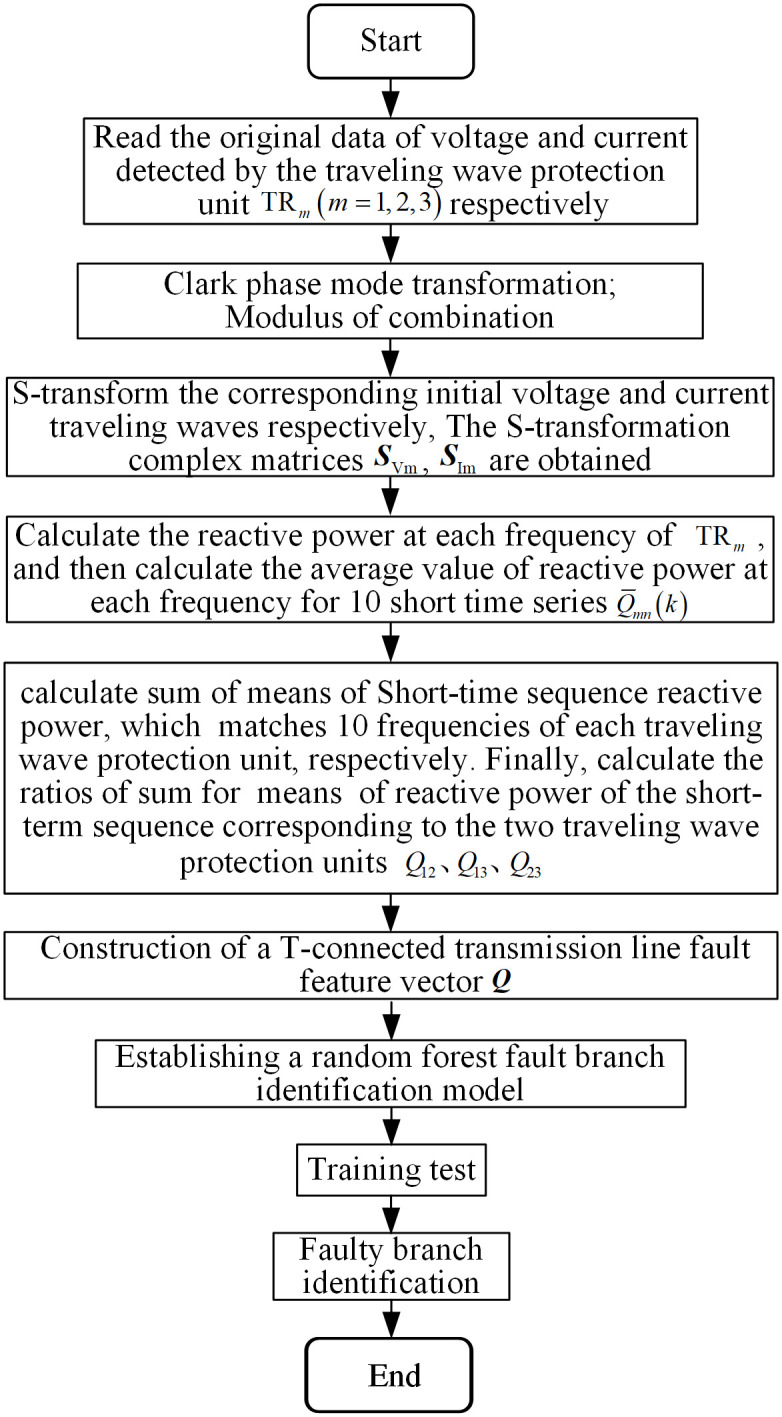
Flow of the fault branch identification algorithm.

In this paper, the initial voltage and current traveling wave data corresponding to the traveling wave protection unit *TR*_*m*_ (*m* = 1, 2, 3) in the 0.1ms time period after the fault are selected at a frequency of 6-15kHz after S-transformation, and calculate the ratio of the mean sum of reactive power under each corresponding time window of each pair of traveling wave protection units. The ratio $Q={\left[\frac{Q_{1\_1}}{Q_{2\_1}},\frac{Q_{1\_2}}{Q_{2\_2}},\cdots ,\frac{Q_{1\_10}}{Q_{2\_10}},\frac{Q_{1\_1}}{Q_{3\_1}},\frac{Q_{1\_2}}{Q_{3\_2}},\cdots ,\frac{Q_{1\_10}}{Q_{3\_10}},\frac{Q_{2\_1}}{Q_{3\_1}},\frac{Q_{2\_2}}{Q_{3\_2}},\cdots ,\frac{Q_{2\_10}}{Q_{3\_10}}\right]}_{1\times 30}$ of the multi-scale initial traveling wave reactive power mean value and specific fault feature vectors are constituted so as to characterize the fault characteristics of the T-connection transmission line. A sample set that can characterize the fault characteristics including randomly simulate the faults with different fault types, different transition resistances, different fault distances and different fault initial angles in each branch of T-connected transmission lines. The flowchart of the fault branch identification algorithm is shown in Fig 11.

## 4 Simulation and experiment

**Fig 12:**
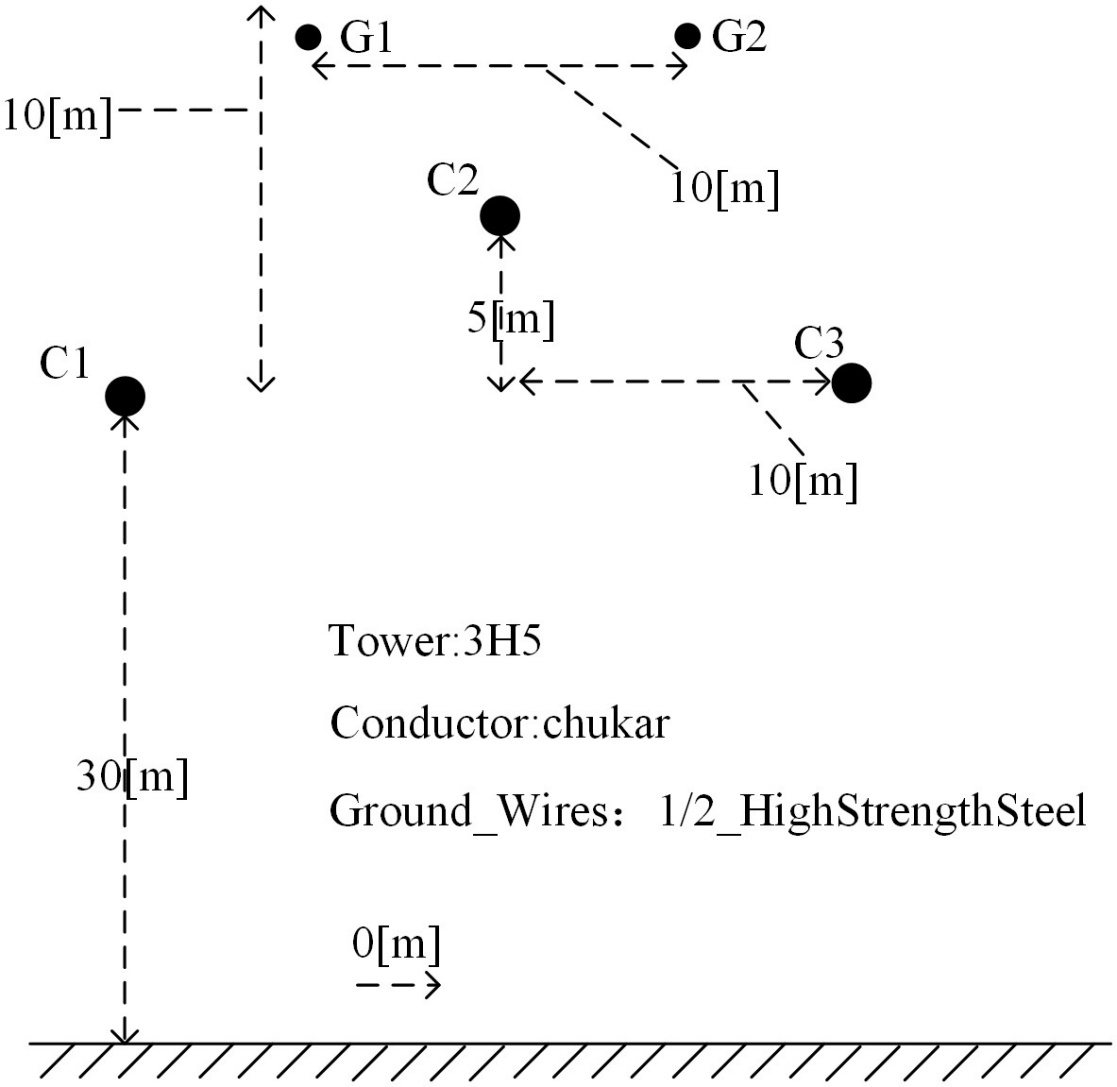
Power line configuration.

PSCAD/EMTDC electromagnetic transient simulation software is used to establish the simulation model of 500kV T-connection transmission line shown in Fig 1. The model of transmission line adopts the frequency related distributed parameter model that can accurately reflect the transient and harmonic response. The tower type of line is: 3H5 [26]. The configuration of transmission line is shown in Fig 12. Among them, the parameters of transmission line are shown in Table 1 [28, 34]. The stray capacitance of busbar is set as *C*_*m*_ = 0.001*μF*. The simulation sampling frequency is 200kHz, and the length of each branch are as followings: AO = 300km, BO = 200km, CO = 150km, AD = 170km, BE = 150km, CF = 180km. All symbols and terms involved in this paper are explained, as shown in Table 2.

**Table 1:** Parameters of the system.

Wire Type	Parameters	Value
Phase Line	Wire radius /m	0.0203454
	DC Resistor /(Ω/km)	0.03206
Ground Line	Wire radius /m	0.0055245
	DC Resistor /(Ω/km)	2.8645

**Table 2:** Explanation of symbols or terms.

Symbols or Terms	Description
AG, BG, CG	Single-phase grounding fault
AB, AC, BC	Two-phase short circuit fault
ACG, ABG, BCG	Two-phase grounding fault
ABC	Three-phase short circuit fault
faults inside the area	As shown in Fig 1, the faults occurred on branches named AO, BO and CO in T-connected transmission line area.
faults outside the area	As shown in Fig 1, the faults occurred on branches AD, BE and CF outside the T-connected transmission line area.

### 4.1 Training sample data

In order to verify the validity and reliability of the algorithm proposed in this paper in the identification of faulty branches, 6 branches of the T-connection transmission line are tested under different fault initial angles, transitional resistances, fault types and fault distances. Each branch is simulated by 5 groups of faults. And a total of 120 sets of fault feature vectors are obtained to form a training sample set for fault branch identification.

### 4.2 Establishment and testing of fault branch identification model

**Fig 13:**
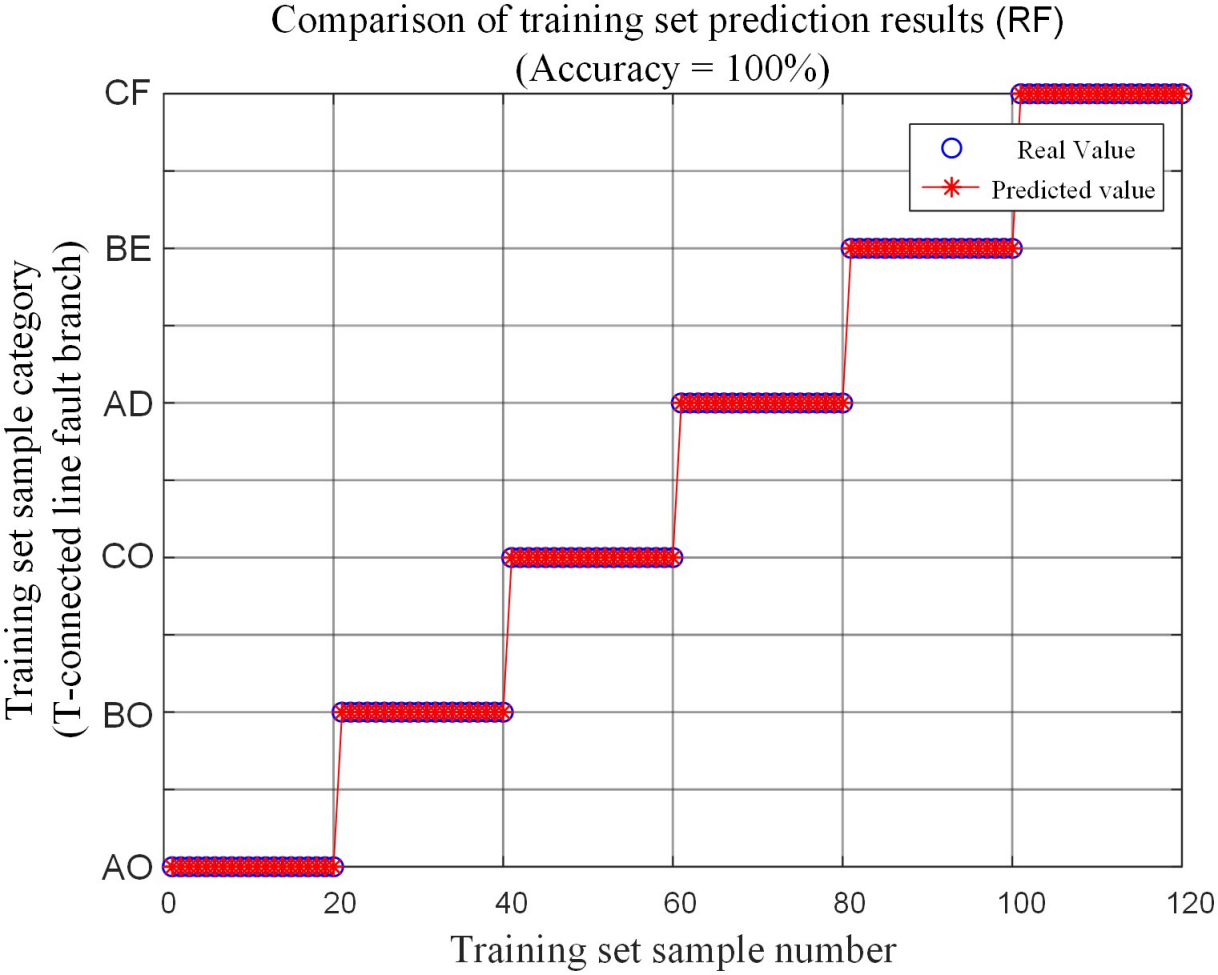
Comparison of training set prediction results.

Input the fault characteristic training samples into the random forest neural network for training, and obtain a trained random forest T-connection transmission line intelligent fault identification model. Then input the fault training samples into the trained random forest fault identification model for testing, and the comparison of the prediction results is shown in Fig 13.

It can be seen from Fig 13 that the accuracy rate of the test results of the training samples in the recognition model is 100%. Selection of training sample failure parameters is shown in Table 3.

**Table 3:** Training sample failure condition parameter selection table.

Fault situation	Parameter selection
Initial angle/degree of failure	0, 5, 25, 45, 60, 90, 100, 120, 145
Transition resistance /Ω	Any choice between 0 and 500 Ω
fault type	AG, BG, CG, AB, AC, BC, ACG, ABG, BCG, ABC
Fault distance /km	The fault location is arbitrarily selected in each branch.

### 4.3 Test sample analysis

In order to further verify the reliability of the proposed algorithm to identify faulty branches, this section simulates six branches inside and outside the protection zone of the T-connection transmission line under different initial fault angles, transitional resistances, fault types, and fault distances. Then four groups test sample sets of faults are obtained, which are different from the training samples Each sample set contains 24 sets of fault feature vectors. Finally, the test sample sets are respectively input into the random forest intelligent fault branch identification model for testing.

#### 4.3.1 Fault initial angle test

**Fig 14:**
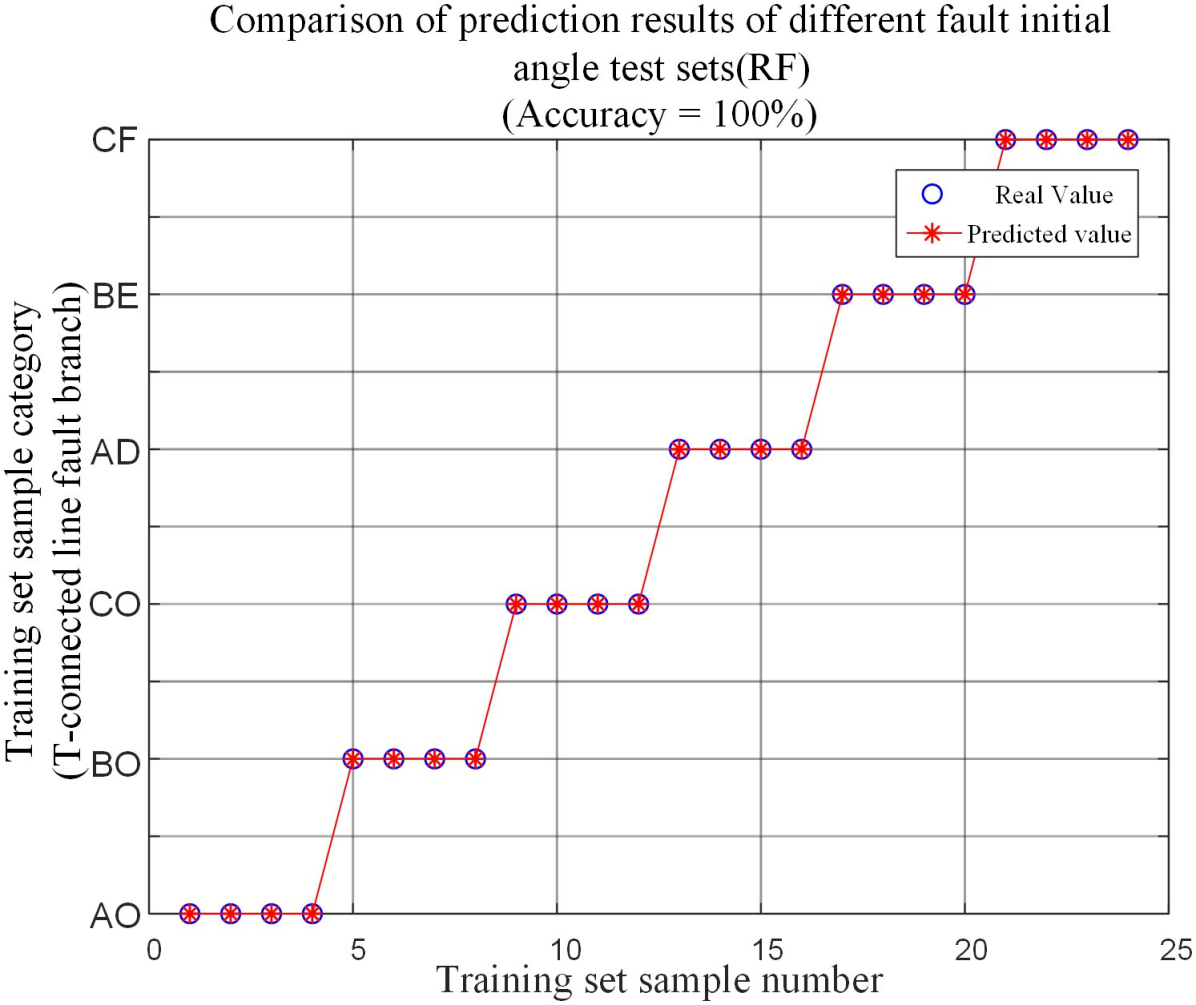
Comparison of different fault initial angle fault test set predictions.

The test simulation samples obtained by different initial fault angles testing are input into the random forest fault branch identification model for testing. The prediction results are shown in Table 4 and Fig 14.

**Table 4:** Simulation results of different initial angle test sets.

Fault branch	Fault initial angle/degree	Fault type	Distance from point O / km	Transitional resistance / Ω	identification result
AO	5	ACG	160	300	AO
	45				AO
	90				AO
	120				AO
BO	5	ABG	140	100	BO
	60				BO
	100				BO
	120				BO
CO	25	AG	70	200	CO
	60				CO
	100				CO
	120				CO
AD	5	AC	410	300	AD
	60				AD
	90				AD
	120				AD
BE	5	BCG	280	50	BE
	45				BE
	100				BE
	120				BE
CF	5	ABC	250	300	CF
	45				CF
	90				CF
	145				CF

When different initial angle faults occur in transmission lines, the algorithm can accurately identify the specific fault branches of the line. The proposed algorithm is not affected by the initial angle of fault.

#### 4.3.2 Transitional resistance tests

The test simulation samples of obtained by different transitional resistance obtained by simulation are input into the random forest fault branch identification model for testing. The prediction results are shown in Table 5.

**Table 5:** Simulation results of different transitional resistance fault test sets.

Fault branch	Transitional resistance / Ω	Fault initial angle/degree	Distance from point O / km	Fault type	identification result
AO	50	120	100	BG	AO
	100				AO
	200				AO
	500				AO
BO	100	25	120	AC	BO
	200				BO
	300				BO
	400				BO
CO	50	60	120	ABC	CO
	200				CO
	300				CO
	500				CO
AD	100	5	410	AG	AD
	200				AD
	400				AD
	500				AD
BE	100	90	250	ABG	BE
	200				BE
	300				BE
	400				BE
CF	200	45	270	CG	CF
	300				CF
	400				CF
	450				CF

When the transmission line has different transitional resistance faults, the algorithm can accurately identify the specific faulty branch of the lines. The proposed algorithm is not affected by the transitional resistance.

#### 4.3.3 Testing of fault types

The test simulation samples of obtained by different faulty branches obtained by simulation are input into the random forest fault branch identification model for testing. The prediction results are shown in Table 6.

**Table 6:** Simulation results of different fault type test sets.

Fault branch	Fault type	Fault initial angle/degree	Distance from point O / km	Transitional resistance / Ω	identification result
AO	AG	60	140	400	AO
	BC				AO
	ACG				AO
	ABC				AO
BO	BG	25	120	50	BO
	AB				BO
	ABG				BO
	BCG				BO
CO	CG	45	50	300	CO
	BCG				CO
	AC				CO
	ACG				CO
AD	BG	100	370	200	AD
	ACG				AD
	AB				AD
	BC				AD
BE	CG	60	245	500	BE
	AB				BE
	ACG				BE
	ABC				BE
CF	AG	5	200	100	CF
	AC				CF
	ABG				CF
	BCG				CF

When different fault types occur in the transmission line, the algorithm can accurately identify the specific fault branch of the transmission line. The proposed algorithm is not affected by fault types.

#### 4.3.4 Fault distance test

The test simulation samples of obtained by different fault distances obtained by simulation are input into the random forest fault branch identification model for testing. The prediction results are shown in Table 7.

**Table 7:** Simulation results of different fault distance test sets.

Fault branch	Distance from point O / km	Fault initial angle/degree	Fault type	Transitional resistance / Ω	identification result
AO	1	5	BCG	200	AO
	20				AO
	190				AO
	230				AO
BO	1	120	CG	300	BO
	5				BO
	100				BO
	180				BO
CO	1	25	AC	400	CO
	2.5				CO
	60				CO
	130				CO
AD	320	60	ABC	100	AD
	350				AD
	375				AD
	425				AD
BE	210	145	BG	200	BE
	240				BE
	280				BE
	310				BE
CF	170	100	BC	300	CF
	195				CF
	230				CF
	305				CF

When faults occur at different fault distances of transmission line, the algorithm can accurately identify the specific fault branch of the transmission line. The proposed algorithm is not affected by the fault distance.

### 4.4 Algorithm performance test analysis

#### 4.4.1 Test and analysis of anti-CT saturation ability

In order to verify the anti-CT saturation performance of the algorithm proposed, the simulation analysis of CT saturation are made when each branch of the T-connection transmission line fails. The CT saturation of the branch CO in the T-connection transmission line is taken as an example to carry out the simulation of CT saturation. The model adopts a nonlinear time-domain equivalent circuit model with relatively good time-frequency characteristics [35].

**Fig 15:**
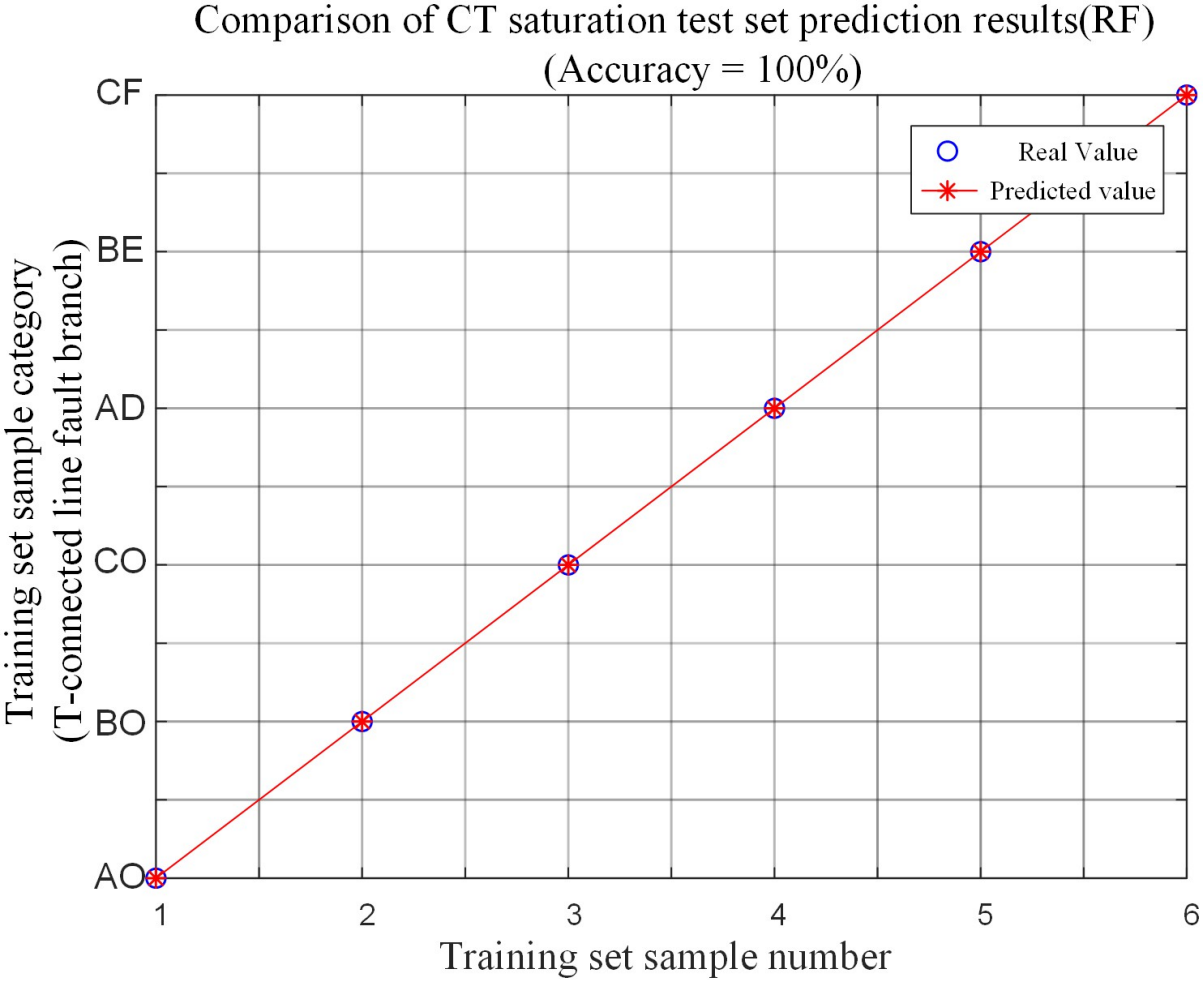
CT saturation test set prediction comparison.

Under the condition of CT saturation on the branch CO in the protection zone of the T-connection transmission line, a group of faults is simulated in each branch of the T-connection transmission line. 6 groups of T-connection transmission line fault characteristic vectors are obtained. Then the fault feature test sample matrix is input into the random forest fault branch identification model for testing. And the test set prediction results are shown in Table 8 and Fig 15 to compare the prediction results corresponding to the fault conditions.

**Table 8:** Simulation results of the test set when internal branch CO of the T-connection transmission line exhibits CT saturation.

Fault branch	Fault type	Fault initial angle/degree	Distance from point O / km	Transitional resistance / Ω	identification result
AO	CG	45	230	300	AO
BO	BC	5	110	400	BO
CO	ACG	120	45	100	CO
AD	AC	60	355	300	AD
BE	BG	120	300	50	BE
CF	ABC	25	215	200	CF

It is seen from the analysis of the above chart that when CT saturation occurs in the branch CO in the protection zone of T-connection transmission line, The algorithm can identify fault branches with 100% accuracy. And it is less affected by CT saturation.

#### 4.4.2 Noise impact analysis test analysis

**Fig 16:**
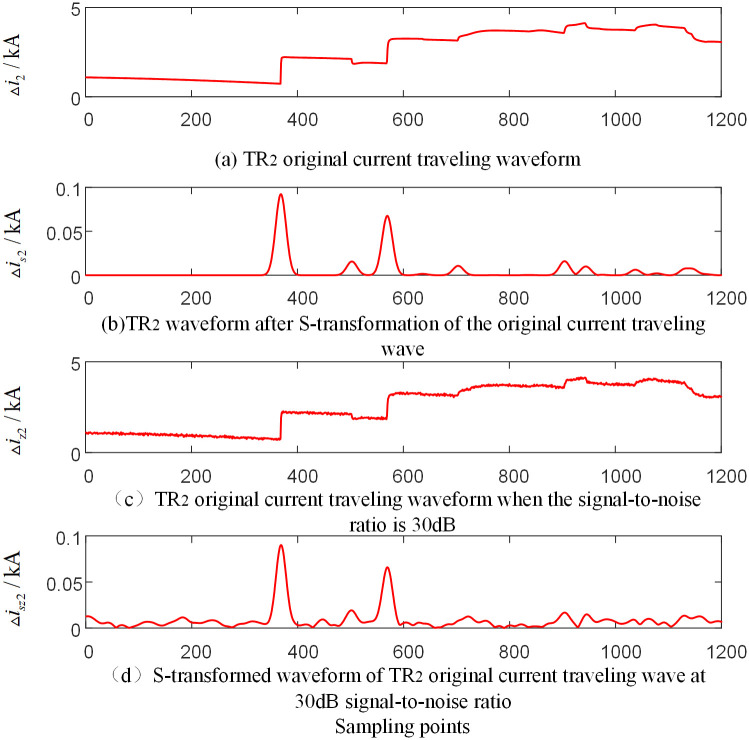
T-connection transmission line traveling wave protection unit *TR*_2_ measured current-dependent waveform when internal branch BO occur fault.

**Fig 17:**
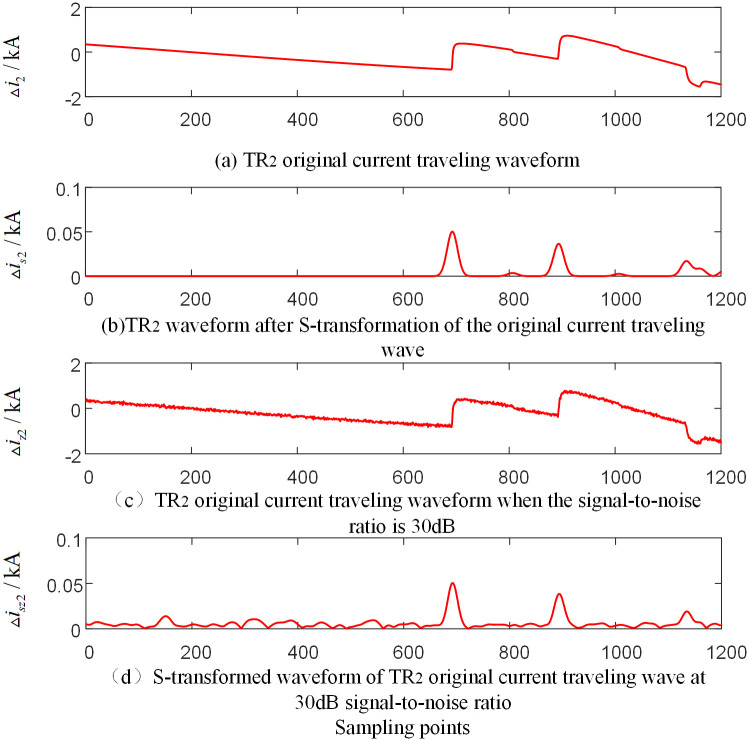
T-connection transmission line traveling wave protection unit *TR*_2_ measured current-dependent waveform when internal branch AD occur fault.

In order to verify the reliability of the algorithm under the influence of noise, noise is added to the voltage and current signals measured by each traveling wave protection unit *TR*_*m*_ (*m* = 1, 2, 3) of the T-connection transmission line. And the signal-to-noise ratio (SNR) is 30dB~70dB. Fig 16 is the current-related traveling wave waveform measured by the traveling wave protection unit *TR*_2_ when a fault occurs on branch BO of the T-connection transmission line. And Fig 17 is the current-related traveling wave waveform measured by the traveling wave protection unit *TR*_1_ when a fault occurs on branch named BE outside the protection zone of the T-connection transmission line. And the current traveling wave measured by the traveling wave protection unit *TR*_1_ and *TR*_2_ are taken as an example under the conditions that the signal-to-noise ratio is 30dB and the frequency after S-transformation is 15kHz.

**Fig 18:**
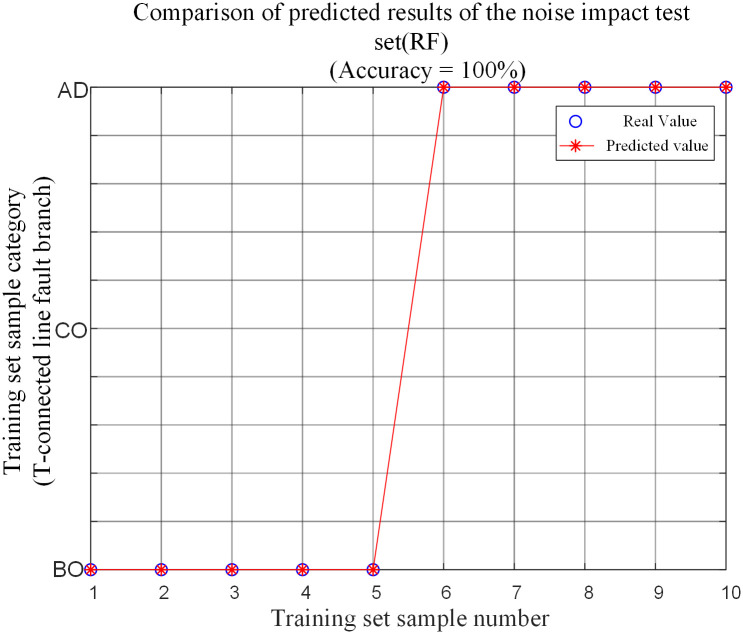
Comparison of noise impact test set predictions.

A fault condition different from the training samples is selected for the branch named AO in the protection zone and the branch named BE outside the protection zone. The noise is added to the voltage and current signals. The signal-to-noise ratios (SNRs) are set as 30dB, 40dB, 50dB, 60dB, and 70dB respectively. 10 groups of T-connection transmission line fault feature vectors are obtained by simulation. And the fault feature test sample matrix is input into the random forest fault branch identification model for testing. As shown in Table 9 and Fig 18, a comparison of the prediction results of the corresponding fault conditions is made.

**Table 9:** Simulation results of test set for internal branch BO and external branch AD in T-connection transmission line under different SNR faults.

Fault branch	SNR/(dB)	Fault type	Fault initial angle/degree	Distance from point O / km	Transitional resistance / Ω	identification result
BO	30	AG	45	100	100	BO
	40					BO
	50					BO
	60					BO
	70					BO
AD	30	ABG	100	385	300	AD
	40					AD
	50					AD
	60					AD
	70					AD

From the results in the above table, the algorithm can identify fault branches with 100% accuracy when the branch BO in the protection zone and the branch AD outside the protection zone under different signal-to-noise ratio faults. Also, this method is less affected by noise.

#### 4.4.3 Data random loss test analysis

The protection device may experience data loss in actual operation. In order to verify the algorithm performance in this case, take the random loss of the initial current traveling wave data measured by the protection unit *TR*_1_ as an example. The branch BO in the protection zone and the branch AD outside the protection zone are selected for simulation analysis, respectively.

**Fig 19:**
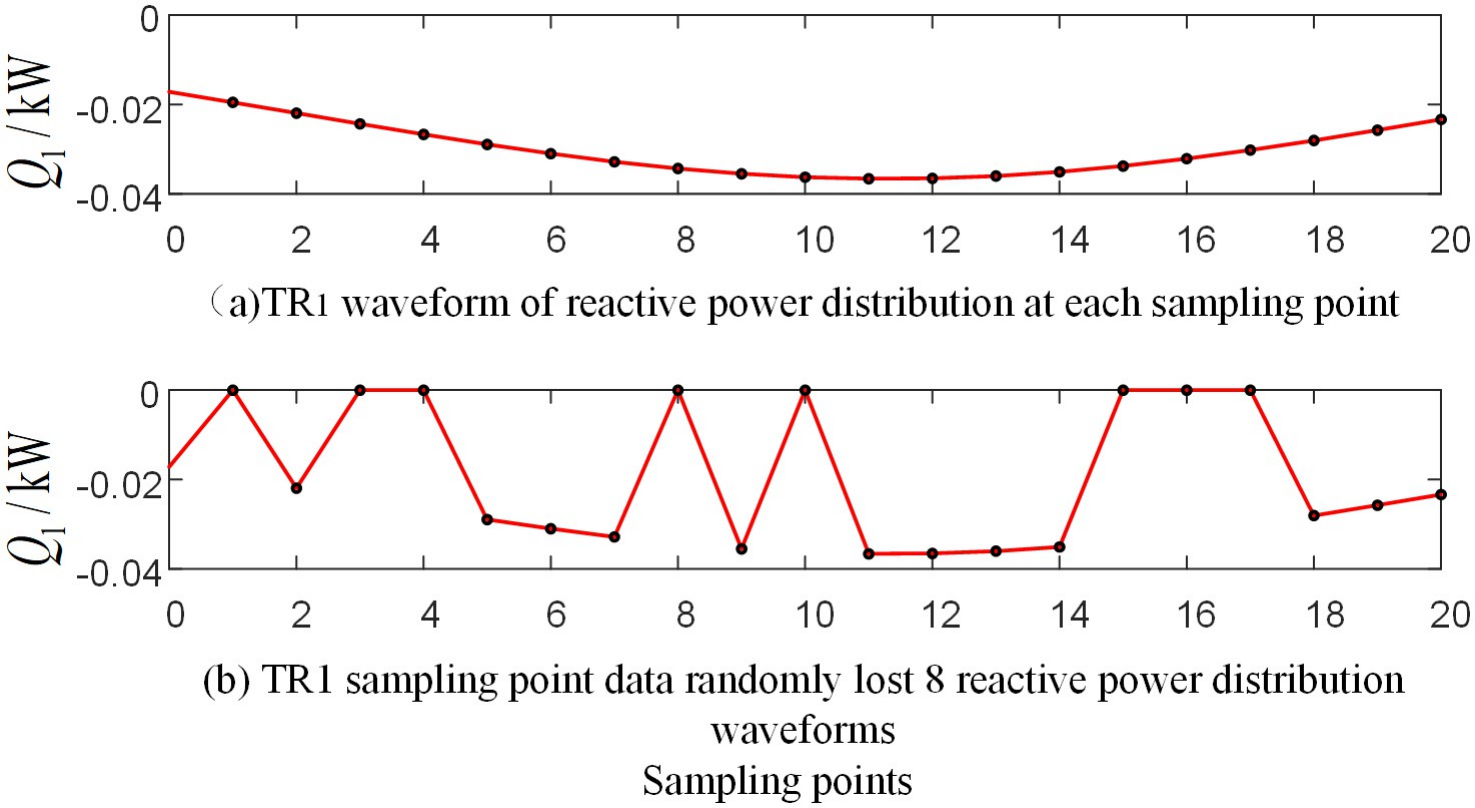
Reactive power at each sampling point corresponding waveform diagram of traveling wave protection unit *TR*_1_ when branch BO occur fault.

**Fig 20:**
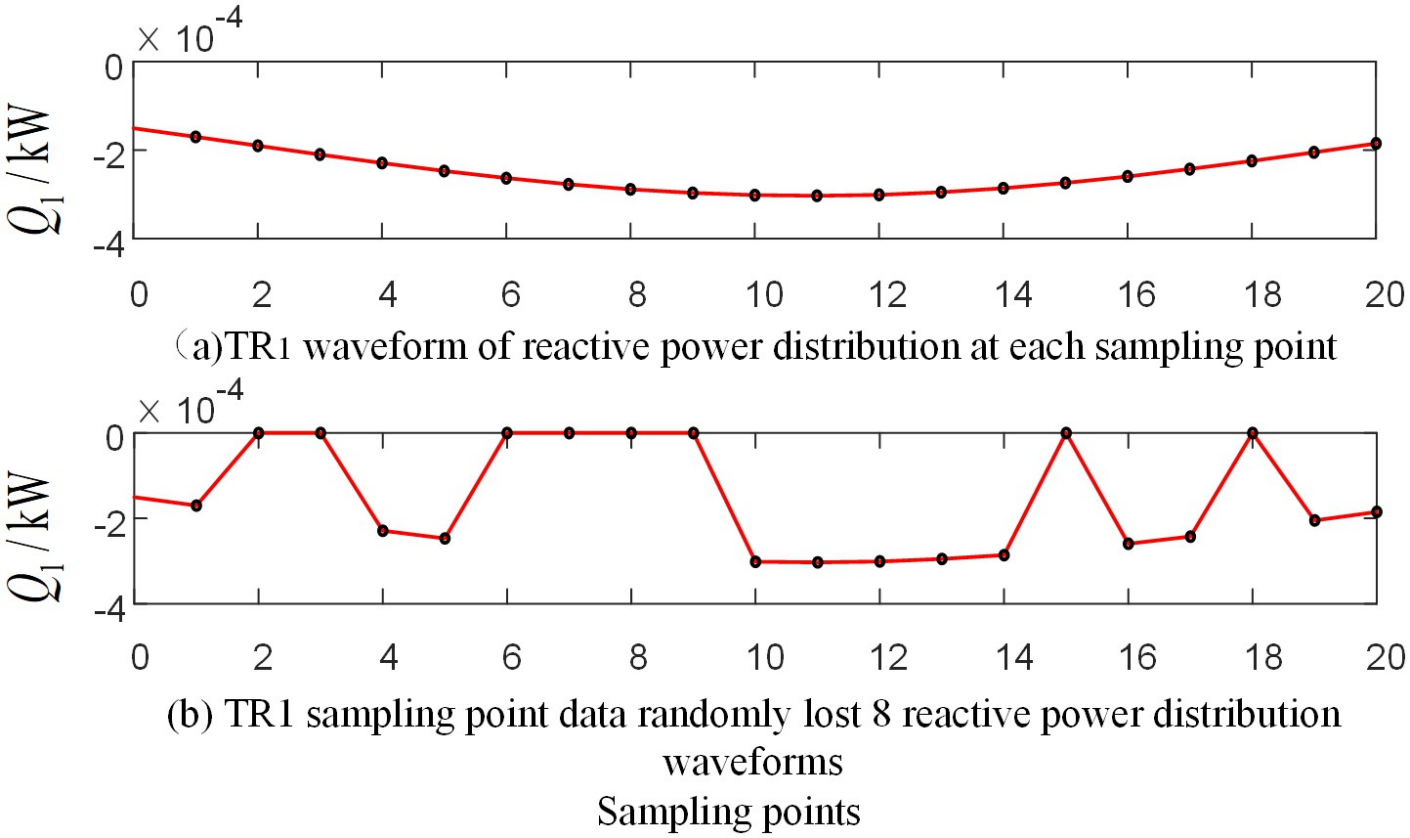
Reactive power at each sampling point corresponding waveform diagram of traveling wave protection unit *TR*_1_ when branch AD occur fault.

Fig 19 shows the relevant waveform of the reactive power distribution after the random loss of data near the initial current traveling wave head when an ACG fault occurs on branch AO in the protection zone at a distance of 120 km from point O. Fig 20 shows the relevant waveform of the reactive power distribution after the random loss of data near the initial current traveling wave head when the BG fault occurs at a distance of 270km from point O on branch CF outside the protection zone. Take the measurement data *TR*_1_ randomly missing 8 sampling points in the data window at a frequency of 15kHz after S-transformation as an example.

**Fig 21:**
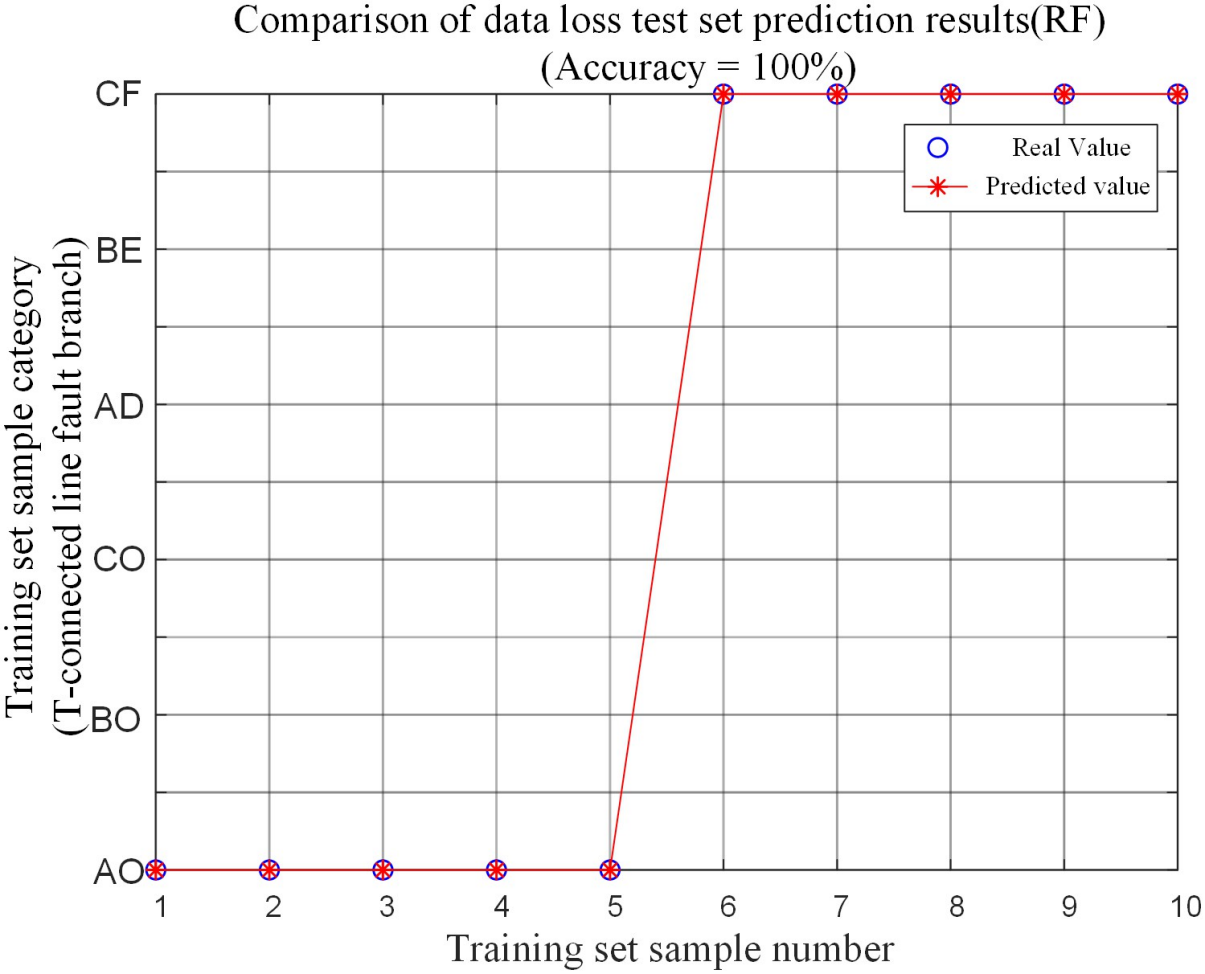
Comparison of data loss test set predictions.

The fault feature test sample is input into the random forest fault branch identification model for testing. And the prediction results are shown in Table 10 and Fig 21 to make a comparison of the prediction results of the corresponding fault conditions.

**Table 10:** Test set simulation verification results for the internal branch AO and external branch CF data randomly lost.

Fault branch	Number of randomly lost data/ one	Fault type	Fault initial angle/degree	Distance from point O / km	Transitional resistance / Ω	identification result
AO	0	ACG	120	120	300	AO
	2					AO
	4					AO
	6					AO
	8					AO
CF	0	BC	25	270	200	CF
	2					CF
	4					CF
	6					CF
	8					CF

The analysis shows that the algorithm can also accurately identify the branch where the fault is located when a fault occurs on the branch inside and outside the protection zone of the T-connection transmission line and the sampling point data near the traveling wave head is randomly lost.

## 5 Discussion

### 5.1 Comparative analysis with traditional algorithm

For algorithm performance, most traditional fault identification algorithms of T-connection line can not analyze the performance of the algorithm under the conditions of noise, CT saturation and data loss. And they can not identify faults accurately in extreme cases. However, under the conditions of noise, CT saturation and data loss, our method could identify faults well.

In terms of algorithm speed, the data window length of the two fault branch identification algorithms proposed in this paper is 0.1ms. Compared with the data window length (20ms or10ms) by full-cycle (half-cycle) Fourier algorithm, our data window length were shorten. And its action speed of the algorithm was improved, which is higher than that of traditional power frequency T-connection line fault identification algorithm.

As for fault identification accuracy, the traditional T-connection line fault identification algorithm can only identify the faults inside and outside the area. And the effects of algorithm are easily influenced by other variables. But the proposed algorithm can not only identify the faults inside and outside the area, but also accurately identify the specific branches of the faults inside and outside the T-connection line.

### 5.2 Analysis of fault diagnosis results of different neural networks

Because different neural networks have different recognition accuracy for the same sample set. In order to analyze the fault diagnosis performance of different neural networks under the proposed algorithm fault sample set, the above training set test sample sets are input into SVM, PNN and RF for testing, respectively. The fault recognition accuracy of different neural networks is shown in Table 11. The calculation process of fault identification accuracy of different sample sets is as follows:

$$Identification\;accuracy=\frac{Identification\;number\;of\;test\;samples}{Total\;number\;of\;test\;samples}\times 100\%$$

**Table 11:** Analysis of fault identification effect of different neural networks.

neural network		SVM	PNN	RF
Test sample(Number of groups)				
Training sample(120)		97.5%	98.33%	100%
Test sample	Different fault types(24)	100%	100%	100%
	Different transition resistance(24)	100%	100%	100%
	Different fault distances(24)	100%	100%	100%
	Different fault initial angles(24)	100%	100%	100%
	Data loss(10)	70%	100%	100%
	CT saturation(6)	100%	100%	100%
	Noise influence(10)	100%	90%	100%

It can be seen in the above table that only random forest can achieve accurate identification of training samples among the three networks. In the test samples, the test samples of different fault types, transition resistances, fault distances and fault initial angles can be accurately identified, while the test samples can only be accurately identified by random forest when analyzing the performance of the three categories of the algorithm.

### 5.3 Reliability analysis of algorithm under different voltage levels and line parameters

To analyze the robustness of the proposed algorithm in different test systems, a simulation model of T-connected transmission line with voltage level of 220kV is established by using PSCAD/EMTDC electromagnetic transient simulation software. And lengths of each branch of T-connected line are AO = 150km, BO = 120km, CO = 140km, AD = 80km, BE = 70km and CF = 80km respectively. The training sample is obtained by randomly simulating five groups of faults in four types of faults with different initial angles, transition resistances, fault types and fault distances of six branches of T-connected transmission lines. The training sample set consists of 120 groups of random fault feature vectors. The test sample is a random simulation of four groups of faults different from the training sample by six branches of T-connection line. The test sample set consists of 24 groups of fault feature vectors. The fault feature sample set is input into RF for training and testing, and the predicted results are shown in Table 12. It can be seen that the algorithm proposed in this paper can also realize the reliable diagnosis of fault branch under this voltage level and line parameters.

**Table 12:** Simulation results of random fault test.

Fault branch	Type	Initial angle	Distance from fault o point /km	Transition resistance /Ω	Recognition result
AO	ACG	25	135	400	AO
	BC	60	100	200	AO
	AG	90	80	300	AO
	ABC	120	40	50	AO
BO	BG	5	105	450	BO
	CG	25	75	100	BO
	BC	100	45	50	BO
	ACG	145	15	200	BO
CO	BG	25	120	300	CO
	BCG	60	95	500	CO
	AC	90	60	400	CO
	ABC	100	5	100	CO
AD	AG	5	220	50	AD
	ACG	45	205	100	AD
	BC	60	180	400	AD
	ABC	120	165	100	AD
BE	CG	25	180	400	BE
	ACG	45	165	100	BE
	BCG	100	140	300	BE
	ABC	120	125	50	BE
CF	AG	25	210	500	CF
	AB	45	190	50	CF
	ABG	90	160	300	CF
	BC	120	145	100	CF

## 6 Conclusion

In this paper, a new fault identification method for T-connection transmission lines is proposed based on multi-scale traveling wave reactive power and random forest network. The characteristics of initial traveling wave reactive power of T-connection transmission line within and outside the fault area are analyzed. Through a large number of simulation experiments, the feasibility of the fault identification method is verified. Theoretical and simulation results show as followings:

1. The proposed fault identification algorithm can quickly and accurately identify the specific fault branch of the T-connection transmission line under various working conditions, and basically overcome the influence of factors such as transitional resistance and fault distance.
2. In terms of algorithm performance, the proposed algorithm can accurately identify the faulty branch under the influence of data information loss, CT saturation and noise (30-70dB), showing good performance and anti-interference ability.
3. In terms of fault identification accuracy, the proposed algorithm can not only identify the faults inside and outside the protection zone, but also can accurately identify the specific branch of the fault, while the traditional T-connection transmission line fault identification algorithm can only identify the faults inside and outside the area, and the recognition effect of some algorithms is easy affected by other variables.

## Supporting information

S1 Fig (TIF)

S1 File Other sample set. (XLSX)

S2 File Test sample set. (XLSX)

S3 File Train sample set. (XLSX)
