# Supplementary figures and images for: A new method for fault identification of T-connection transmission line based on multi-scale traveling wave reactive power and random forest
Source: PLoS One. 2023 Aug 18;18(8):e0284937. doi: 10.1371/journal.pone.0284937 (PMC10437885; doi:10.1371/journal.pone.0284937)

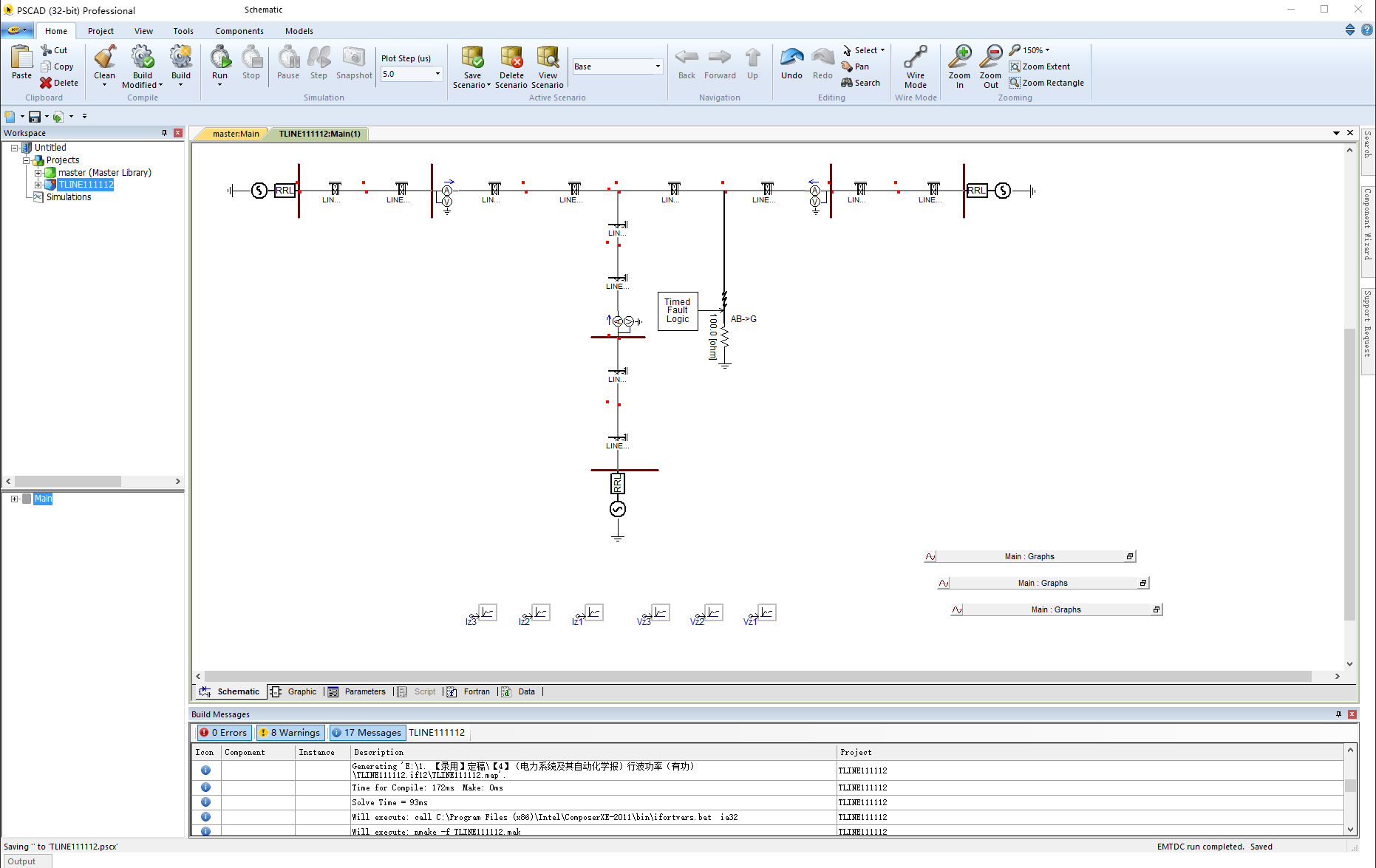

Supplement: S1 Fig — (TIF) [file pone.0284937.s001.tif]
